# Mature neurons from iPSCs unveil neurodegeneration-related pathways in mucopolysaccharidosis type II: GSK-3β inhibition for therapeutic potential

**DOI:** 10.1038/s41419-024-06692-9

**Published:** 2024-04-29

**Authors:** Tzu-Yu Chen, Shuan-Pei Lin, De-Fong Huang, Hsien-Sung Huang, Feng-Chiao Tsai, Li-Jen Lee, Hsiang-Yu Lin, Hsiang-Po Huang

**Affiliations:** 1 Graduate Institute of Medical Genomics and Proteomics, National Taiwan University College of Medicine, Taipei, Taiwan; 2 Department of Medicine, MacKay Medical College, New Taipei City, Taiwan; 3 Department of Pediatrics, MacKay Memorial Hospital, Taipei, Taiwan; 4 Graduate Institute of Brain and Mind Sciences, National Taiwan University College of Medicine, Taipei, Taiwan; 5 Graduate Institute of Pharmacology, National Taiwan University College of Medicine, Taipei, Taiwan; 6 Department of Internal Medicine, National Taiwan University Hospital, Taipei, Taiwan; 7 Graduate Institute of Anatomy and Cell Biology, National Taiwan University College of Medicine, Taipei, Taiwan; 8 Neurobiology and Cognitive Science Center, National Taiwan University, Taipei, Taiwan

**Keywords:** Induced pluripotent stem cells

## Abstract

Mucopolysaccharidosis (MPS) type II is caused by a deficiency of iduronate-2-sulfatase and is characterized by the accumulation of glycosaminoglycans (GAGs). Without effective therapy, the severe form of MPS II causes progressive neurodegeneration and death. This study generated multiple clones of induced pluripotent stem cells (iPSCs) and their isogenic controls (ISO) from four patients with MPS II neurodegeneration. MPS II-iPSCs were successfully differentiated into cortical neurons with characteristic biochemical and cellular phenotypes, including axonal beadings positive for phosphorylated tau, and unique electrophysiological abnormalities, which were mostly rescued in ISO-iPSC-derived neurons. RNA sequencing analysis uncovered dysregulation in three major signaling pathways, including Wnt/β-catenin, p38 MAP kinase, and calcium pathways, in mature MPS II neurons. Further mechanistic characterization indicated that the dysregulation in calcium signaling led to an elevated intracellular calcium level, which might be linked to compromised survival of neurons. Based on these dysregulated pathways, several related chemicals and drugs were tested using this mature MPS II neuron-based platform and a small-molecule glycogen synthase kinase-3β inhibitor was found to significantly rescue neuronal survival, neurite morphology, and electrophysiological abnormalities in MPS II neurons. Our results underscore that the MPS II-iPSC-based platform significantly contributes to unraveling the mechanisms underlying the degeneration and death of MPS II neurons and assessing potential drug candidates. Furthermore, the study revealed that targeting the specific dysregulation of signaling pathways downstream of GAG accumulation in MPS II neurons with a well-characterized drug could potentially ameliorate neuronal degeneration.

## Introduction

Mucopolysaccharidoses (MPS) are inherited lysosomal storage diseases caused by mutations leading to GAG accumulation and cellular dysfunction [1]. Mucopolysaccharidosis type II (MPS II), also known as Hunter syndrome [2], is the most frequent MPS. It is an X-linked recessive disorder predominantly affecting males, resulting from mutations in the *IDS* gene, which encodes the lysosomal enzyme iduronate-2-sulfatase (IDS). The enzyme hydrolyzes sulfate groups in dermatan sulfate (DS) and heparan sulfate (HS) molecules [3], whose accumulation in MPS II cells disrupts cellular homeostasis and causes organ abnormalities.

The main treatment of MPS II, enzyme replacement therapy (ERT), cannot cross the blood-brain barrier (BBB) [4]. This leaves the severe-form MPS II, which affects the central nervous system, without an effective treatment [5], underscoring the urgency for more dependable models and therapies.

Our study aimed to comprehend the molecular pathogenesis of neurodegeneration and identify potential small-molecule therapeutic candidates. We generated and validated multiple human iPSC clones in four patients with severe-form MPS II, and created mutation-corrected isogenic control (ISO)-iPSCs. These were differentiated into cortical neurons to analyze their biochemical, cellular abnormalities, and electrophysiological malfunctions. Critically, we evaluated the therapeutic effects of various chemicals/drugs in MPS II neurons, targeting dysregulated pathways.

## Results

### Generation of MPS II-iPSCs and their ISO-iPSC clones

We generated MPS II-iPSCs from four severe-form MPS II patients’ peripheral blood mononuclear cells (PBMCs) using Sendai virus reprogramming. Three optimal clones per patient were selected (Supplementary Fig. 1), exhibiting high pluripotency marker gene expression, silenced Sendai viral genes, normal male karyotypes, and original MPS II mutations (Supplementary Fig. 1A–C). These MPS II-iPSCs also uniformly expressed alkaline phosphatase, TRA-1-60, and NANOG (Supplementary Fig. 1D). Their pluripotency was confirmed through both embryoid body-based differentiation and teratoma formation assays (Supplementary Fig. 1E).

Mutation-corrected ISO-iPSC clones were generated from MPS II-iPSCs through genomic editing, validated by Sanger sequencing (Supplementary Fig. 1F), including two clones each for Patients 1 and 4. NANOG and OCT4 expression confirmed their pluripotency (Supplementary Fig. 1G). The control group comprised these four ISO-iPSC clones and three male healthy control (HC)-iPSC clones. The iPSC clone usage in each experiment was detailed in Supplementary Table 1.

### MPS II-iPSCs manifested characteristic MPS II phenotypes

**Fig. 1:**
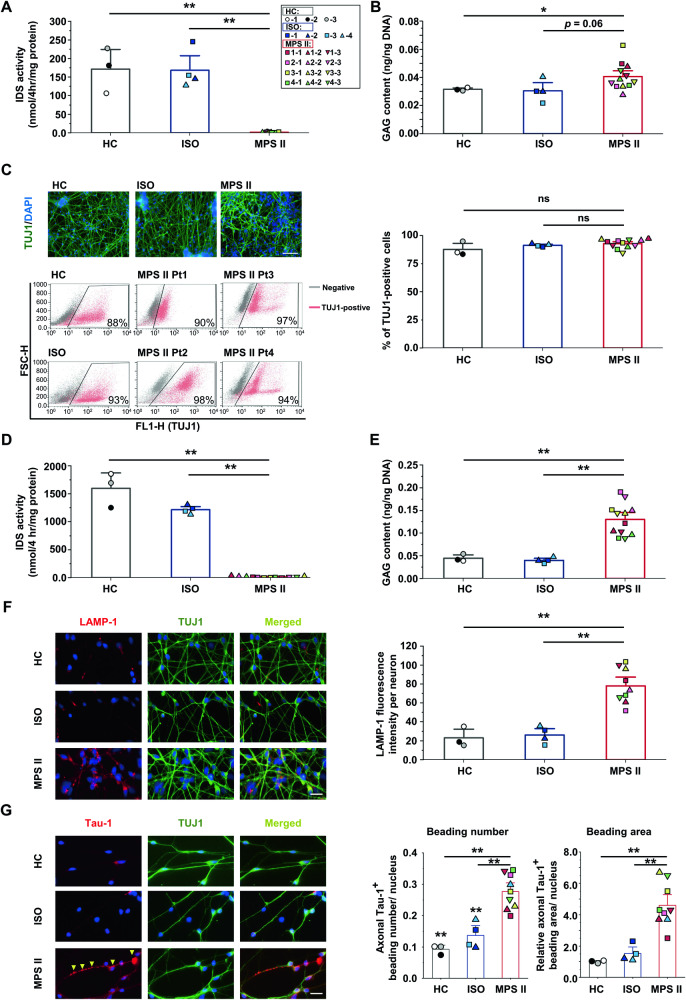
MPS II-iPSCs and the derived cortical neurons recapitulated the pathophysiological features of MPS II. **A** Deficient IDS activity in MPS II-iPSC clones compared with HC- and ISO-iPSC clones. Each color represents for a unique person. Details of cell clones are provided in the top right corner. **B** The GAG content in MPS II-iPSC clones was significantly higher than in both control groups. **C** Representative IF images (upper panels) and flow cytometry results (lower panels) of TUJ1-positive (green) cortical neurons, which were differentiated from the control and MPS II-iPSC clones for four weeks. Nuclei were counterstained by DAPI (blue); Scale bar: 200 μm. **D** Deficient IDS activity in four-week MPS II neurons compared to HC and ISO counterparts. **E** The GAG content in four-week MPS II neurons was higher than in their HC and ISO counterparts. **F** Double IF microscopy showed a significant increase in LAMP-1 signals (red) in four-week MPS II neurons (left). Nuclei were counterstained by DAPI (blue); Scale bar: 20 μm. The histogram (right) shows the quantification results. **G** Double IF microscopy (left) showed increased Tau-1-positive axonal beading number and area in four-week MPS II TUJ-1-positive neurons compared with control neurons. The arrows indicate beading sites. The histograms show the quantification results of the axonal beading number and area (right) using ImageJ software. DAPI counterstain (blue); Scale bar: 20 μm. For **A**–**E**, *N* = 3 HC clones, *N* = 4 ISO clones from 4 patients, and *N* = 12 MPS II clones from 4 patients. For **F**, **G**, *N* = 3 HC clones, *N* = 4 ISO clones from 4 patients, and *N* = 9 MPS II clones from 4 patients. All data are presented as mean ± SEM. *p < 0.05, **p < 0.01, ***p < 0.005, between control (either HC or ISO) and MPS II cells, analyzed by Mann–Whitney U test. Each presented data point originates from independent differentiation. Cell clone details are in Supplementary Table 1.

We assessed MPS II phenotypes in MPS II-iPSCs by comparing their IDS activity and GAG content with HC- and ISO-iPSC clones. MPS II-iPSCs showed lower IDS activity (Fig. 1A) and higher GAG content (Fig. 1B), indicating MPS II phenotypes at the pluripotent stem cell stage.

### MPS II-iPSC-derived neurons exhibited lysosomal/autophagic abnormalities and axonal beadings

To assess the ability of MPS II-iPSCs to generate neuronal populations, we induced neuronal differentiation in these cells for four weeks. Immunofluorescence (IF) microscopy and flow cytometry demonstrated that most differentiated cells expressed TUJ1 (Fig. 1C), an early neuronal marker [6], and some expressed glial cell marker GFAP (Supplementary Fig. 2A). Neuron and glial proportions were consistent across HC-, ISO-, and MPS II-iPSC clones (Fig. 1C, Supplementary Fig. 2A). Notably, the MPS II-iPSC-derived neurons (hereafter MPS II neurons) exhibited significant IDS deficiency and GAG accumulation compared to HC and ISO neurons (Fig. 1D, E).

During neuronal differentiation, IDS activity increased significantly in HC, ISO, and MPS II neurons (Supplementary Fig. 2B), suggesting its role in this process. Only MPS II neurons accumulated GAGs (Supplementary Fig. 2C). At the fourth week, MPS II neurons exhibited an increase in coarse LAMP-1-positive granules (Fig. 1F). Similarly, western blotting at 15 weeks revealed greater LAMP-2 accumulation and a trend towards increased LAMP-1 in MPS II neurons compared to controls (Supplementary Fig. 2D).

Moreover, the 15-week MPS II neurons exhibited reduced autophagy influx compared to HC and ISO neurons, as evidenced by the reduction in the difference of LC3B levels before and after chloroquine treatment (Supplementary Fig. 2E). Morphologically, increased bead-like swellings were noted in the neurites of 4-week MPS II neurons. IF microscopy confirmed Tau-1 positivity in these axonal beadings (Fig. 1G), with most also co-staining for phosphorylated tau (p-tau) (Supplementary Fig. 2F). Hence, MPS II neurons exhibited lysosomal/autophagic abnormalities of MPS II and an unreported neuropathological trait resembling Alzheimer’s disease (AD) [7].

### Anomalies in neurite morphology, cytoskeletal protein expressions, and electrophysiology in long-term cultured MPS II neurons

**Fig. 2:**
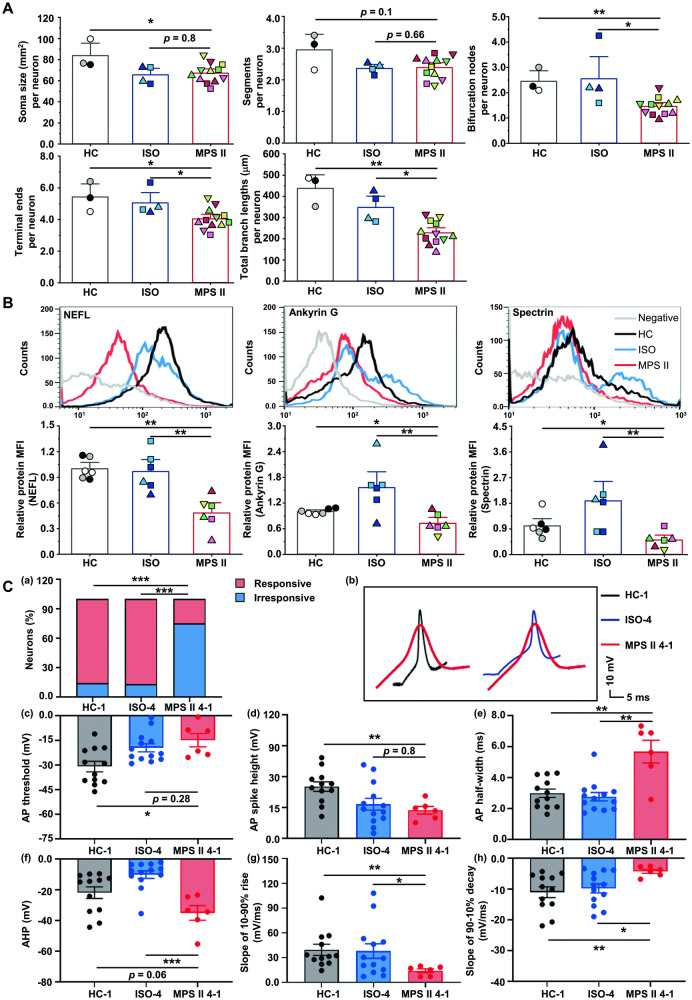
MPS II neurons exhibited anomalies in neurite morphology, *NEFL* gene expression, and action potential. **A** Neurite morphometrics of mature cortical neurons. The 15-week MPS II, HC, and ISO neurons were infected with AAV8-*hSyn-EGFP*. Neuron morphology was reconstructed using Neurolucida. The analyzed neuron morphology parameters included soma size, segments, bifurcation nodes, terminal ends, and total branch lengths. *N* = 3 HC clones, *N* = 4 ISO clones from 2 patients, and *N* = 12 MPS II clones from 4 patients. **B** Flow cytometry analyses demonstrated decreased NEFL, Ankyrin G, and Spectrin protein levels in 15-week MPS II neurons. *N* = 6 HC clones, *N* = 6 differentiations from 2 patients’ 4 ISO clones, and *N* = 6 MPS II clones from 4 patients. **C** Abnormal action potentials (APs) in mature MPS II neurons compared to HC and ISO neurons. The APs of cortical neurons (differentiated for 18 weeks from control and MPS II iPSCs) in response to increasing current steps (−50 to 150 pA) were measured using current-clamp recordings. A decreased response rate to current injections in MPS II neurons compared to those of HC and ISO neurons (upper left panel). *N* = 14 cells of 4 differentiations of HC-1, *N* = 15 cells from 4 differentiations of ISO-4, and *N* = 24 cells from 3 differentiations of MPS II 4-1. ***p < 0.005, analyzed by chi-square test. Representative traces depicted the changes of AP morphology in MPS II neurons compared with HC and ISO neurons. Scale bars: 10 ms (x-axis) and 10 mV (y-axis). Histograms show the differences of AP parameters between MPS II neurons and HC or ISO neurons, including AP threshold, AP spike height, AP half-width, after-hyperpolarization (AHP), the slope of 10–90% rise, and the slope of 90–10% decay. Each data point represents an individual responsive neuron. *N* = 12 cells from 4 differentiations of HC-1, *N* = 13 cells from 4 differentiations of ISO-4, and *N* = 6 cells from 3 differentiations of MPS II 4-1. Data represent mean ± SEM. *p < 0.05, **p < 0.01, ***p < 0.005, analyzed by Mann–Whitney U test. Cell clone details are in Supplementary Table 1.

To characterize neuron morphology, we infected 15-week iPSC-derived neurons with adeno-associated virus (AAV) 8-*hSyn-EGFP* for visualization (Supplementary Fig. 3A). Fluorescence microscopy revealed abnormal neurite morphology in MPS II neurons compared to HC and ISO neurons (Supplementary Fig. 3A). Image analyses revealed fewer bifurcation nodes, terminal ends, and total branch lengths in MPS II neurons (Supplementary Fig. 3A, Fig. 2A). We examined *NEFL*, which encodes the neurofilament light chain, expressed earliest [8] and crucial for neurite outgrowth [9]. *NEFL* mRNA was significantly lower in MPS II neurons, shown by quantitative reverse transcription PCR (qRT-PCR) (Supplementary Fig. 3B). Flow cytometry confirmed reduced NEFL (Fig. 2B), and lower levels of Ankyrin G and spectrin, two proteins essential for neural cytoskeletal integrity and associated with neurodegeneration [10], in MPS II neurons. Whole-cell patch-clamp recording was performed on 18-week iPSC-derived neurons labeled with AAV8-*hSyn-EGFP*. A decreased percentage of responsive neurons was found in MPS II neurons compared to HC neurons (Fig. 2C, Supplementary Fig. 3C) and ISO neurons (Fig. 2C). In responsive MPS II neurons, increasing current steps (−50–150 pA) caused action potential (AP) anomalies, including prolonged half-width, diminished slopes in 10–90% rise and 90–10% decay, and reduced spike height (Fig. 2C, Supplementary Fig. 3C). To further assay the function of neuronal subtypes, the 20-week iPSC-derived neurons were infected with AAV9-*CaMKII α*-*EYFP* and AAV9-*mDlx-GFP* to label excitatory (the majority) and inhibitory neurons (the minor), respectively (Supplementary Fig. 3D). Both excitatory and inhibitory MPS II neurons exhibited abnormal neurite morphology, with fewer bifurcation nodes compared to HC neurons (Supplementary Fig. 3E, F). Specifically, MPS II excitatory neurons had shorter branch lengths, while inhibitory neurons had fewer terminal ends compared to HC neurons (Supplementary Fig. 3E, F). Electrophysiologically, in 22-week MPS II excitatory neurons, there was a slightly reduced percentage of neurons responsive to current injection compared to HC neurons (Supplementary Fig. 3G). They also displayed abnormal AP, with decreased spike height, prolonged half-width, and reduced 10–90% rising slope compared to HC neurons (Supplementary Fig. 3G).

### Identification of dysregulated genes and pathways in MPS II neurons

To explore MPS II neurodegeneration and identify specific gene expression changes, we conducted RNA sequencing on 15-week HC neurons (3 clones) and MPS II neurons (9 clones), identifying 921 upregulated and 649 downregulated differentially expressed genes (DEGs) in MPS II neurons.

**Fig. 3:**
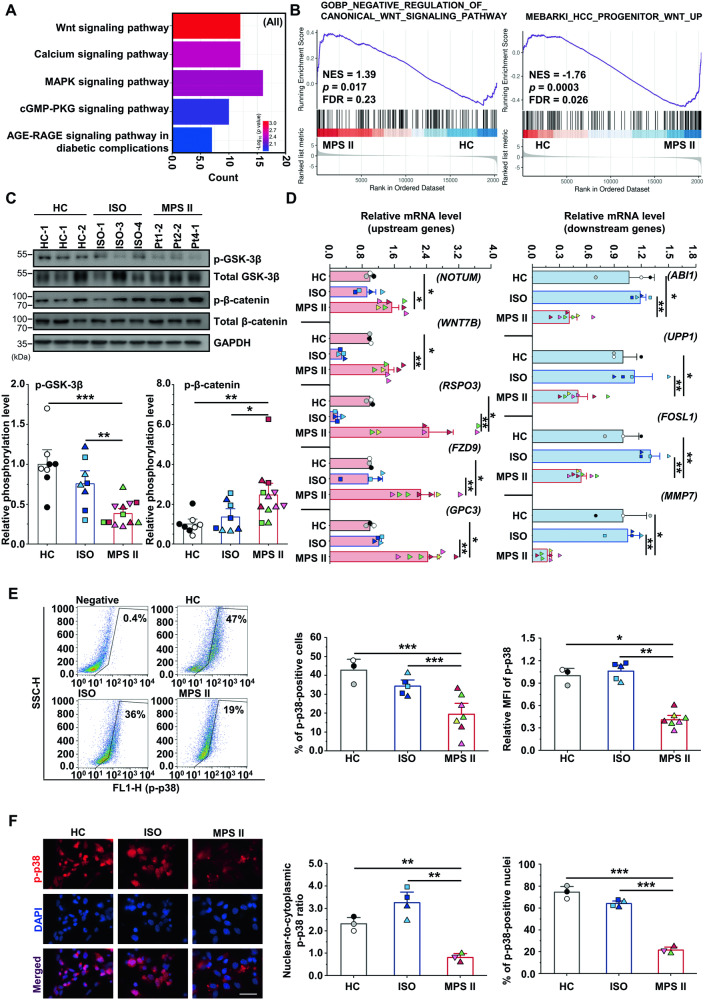
Transcriptome profiling revealed significant alterations in signaling pathways in MPS II neurons. **A** KEGG pathway analysis of DEGs in MPS II neurons compared to HC neurons revealed significant changes in multiple signaling pathways. **B** GSEA of DEGs in MPS II neurons revealed significant enrichment of a gene set involved in negative regulation of the canonical Wnt signaling pathway (left), and a gene set that was negatively correlated with Wnt upregulation (right) in 15-week-old MPS II neurons compared with their HC counterparts. **C** Decreased GSK-3β phosphorylation and increased β-catenin phosphorylation in 15-week-old MPS II neurons compared with control neurons. The upper panel shows a representative western blotting result, comparing the phosphorylation levels of GSK-3β at Ser9 and β-catenin at Ser33/37/Thr41 among HC, ISO, and MPS II neurons. The lower panels depict the relative phosphorylation levels of GSK-3β (left) and β-catenin (right) in different groups of neurons. The levels resulted from normalizing the phosphorylation signals to respective total GSK-3β or β-catenin signals, as well as GAPDH signals, and are presented as mean ratios relative to HC neurons. *N* = 8 differentiations from 2 HC clones, *N* = 8 differentiations from 4 ISO clones, and *N* = 12 differentiations from 6 MPS II patients’ clones. **D** The qRT-PCR analysis confirmed the altered expression of five representative Wnt pathway genes, including five well-known upstream genes (*NOTUM*, *WNT7B*, *RSPO3*, *FZD9*, and *GPC3*) and four downstream genes (*ABI1*, *UPP1*, *MMP7*, and *FOSL1*) in 15-week-old MPS II neurons compared to their control counterparts. *N* = 3 HC clones, *N* = 4 ISO clones, and *N* = 7 differentiations from 4 MPS II patients’ clones. **E** Representative dot plots for the flow cytometry analysis of phosphorylated p38 MAPK-positive neurons. Histograms for the flow cytometry show a decreased percentage of phosphorylated p38 MAPK (p-p38)-positive cells, and a lower mean fluorescence intensity (MFI) of p-p38 MAPK GFP signals in 15-week MPS II cortical neurons compared with their control counterparts. *N* = 3 HC clones, *N* = 5 differentiations from 4 ISO clones, and *N* = 7 differentiations from 4 MPS II patients’ clones. *p < 0.05, **p < 0.01, analyzed using chi-square test for positive cell percentage data, and Mann–Whitney U test for MFI data. **F** Representative IF microscopy images of p-p38 MAPK-positive iPSC-derived neurons. Quantification showed a significantly decreased nuclear-to-cytoplasmic ratio of p-p38 MAPK fluorescence intensity (left histogram), and a lower percentage of nuclei positive for p-p38 MAPK (right histogram) in 15-week MPS II neurons compared to their control counterparts. DAPI counterstain: blue. Scale bar: 20 μm. The intensity of nuclear and cytoplasmic p-p38 signals and the percentage of p-p38 MAPK-positive nuclei were quantified using ImageJ. *N* = 3 HC clones, *N* = 4 ISO clones, and *N* = 3 MPS II patients’ clones. **p < 0.01, ***p < 0.005, analyzed using Welch’s test for fluorescence intensity ratio data, and chi-square test for nuclei positivity percentage data. Data in other panels represent mean ± SEM. *p < 0.05, **p < 0.01, ***p < 0.005, analyzed by Mann–Whitney U test. Cell clone details are in Supplementary Table 1.

Ingenuity Pathway Analysis (IPA) was employed to investigate the DEGs that might influence the neuronal AP, identifying three ion channel-encoding genes: *SCN9A* (sodium channel NaV1.7) [11]*, KCNMB2* [12], and *KCND2* (potassium channels) [13]. The qRT-PCR confirmed their lower expression in MPS II neurons (Supplementary Fig. 3H). Flow cytometry showed lower protein levels of these genes in MPS II neurons (Supplementary Fig. 3I), which might contribute to the AP anomalies. Furthermore, Kyoto Encyclopedia of Genes and Genomes (KEGG) pathway enrichment analysis revealed significant DEG enrichment in several pathways, with Wnt ranking highest, followed by calcium and MAPK signaling pathways in MPS II neurons (Fig. 3A).

Additionally, gene set enrichment analysis (GSEA) demonstrated genes in MPS II neurons significantly enriched in negative regulation of Wnt pathway (Fig. 3B, left) and downregulation of WNT3A-responsive genes (Fig. 3B, right). KEGG pathway analysis revealed that upregulated genes in Wnt signaling were mostly upstream regulators, while downregulated genes were downstream TCF/LEF targets (Supplementary Fig. 4A). These suggested attenuated intracellular Wnt signaling in MPS II neurons, with upstream regulator upregulation possibly compensating for this attenuation. Supporting this, western blotting demonstrated decreased phosphorylated GSK-3β (Ser9, p-GSK-3β, inactive), and increased β-catenin phosphorylation (Ser33/37/Thr41, p-β-catenin, inactive) in MPS II neurons (Fig. 3C). It also revealed less nuclear and more cytosolic non-phosphorylated β-catenin (active) in MPS II neurons (Supplementary Fig. 4B). Next, we analyzed nine established Wnt signaling genes from the DEGs (Supplementary Fig. 4C) and literature [14–16] using qRT-PCR, confirming upregulation of five upstream genes (*NOTUM*, *WNT7B*, *RSPO3*, *FZD9*, and *GPC3* [14]) and downregulation of four downstream genes (*ABI1* [15], *UPP1* [16], *FOSL1*, and *MMP7*) in MPS II neurons (Fig. 3D). Additionally, the 15-week iPSC-derived neurons transduced with Wnt reporter construct-containing lentiviruses showed significantly lower Wnt reporter activity in MPS II neurons compared to control neurons (Supplementary Fig. 4D). These findings collectively confirmed reduced Wnt/β-catenin signaling in MPS II neurons.

IPA showed that p38 MAPK signaling was significantly altered in MPS II neurons (Supplementary Table 2), consistent with dysregulated MAPK pathway in KEGG pathway enrichment analysis (Fig. 3A) and the suppressed fibroblast growth factor (FGF) signaling disclosed by GSEA (Supplementary Fig. 4E). Concordantly, flow cytometry (Fig. 3E) showed reduced phosphorylated p38 (p-p38) MAPK in 15-week MPS II neurons, both in cell percentage and fluorescent intensity. IF microscopy showed a lower nuclear-to-cytoplasmic p-p38 ratio and fewer p-p38 MAPK-positive nuclei in MPS II neurons (Fig. 3F). This trend was also observed in total p38 MAPK (Supplementary Fig. 4F). Furthermore, MPS II neurons responded less to Wnt activator WNT3A and p38 MAPK activator bFGF2, showing decreased activation of downstream genes (*AXIN2, MYC*, and *MMP7* for WNT3A [17]; *FOSL1* [18]*, CCDC115* [19], and *OTX2* [20] for FGF2) (Supplementary Fig. 4G, H).

### Dysregulated calcium homeostasis in MPS II neurons and the possible mechanisms

**Fig. 4:**
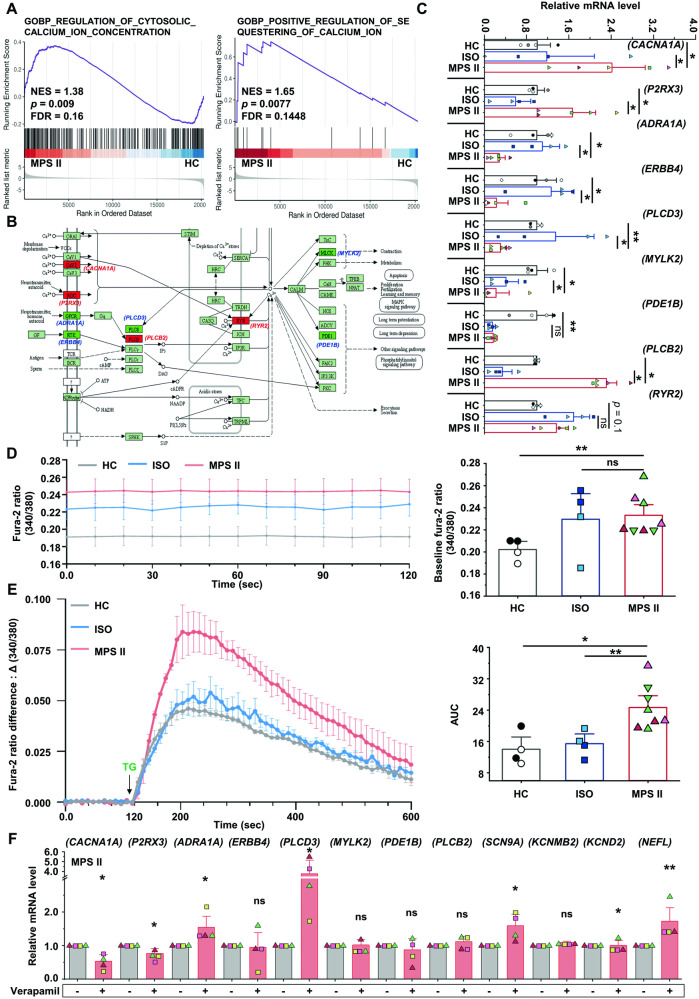
Altered expressions of calcium signaling-related genes and intracellular calcium levels in MPS II neurons. **A** GSEA of RNA-seq data indicated significant enrichment of the genes regulating cytosolic calcium ion concentrations (left panel) and positive regulation of calcium ion sequestering (right panel) in MPS II neurons (*N* = 9) compared with HC neurons (*N* = 3). **B** Comparative analysis of RNA-seq data between the 15-week MPS II and HC neurons highlighted a change in the calcium signaling pathway (hsa04020 in KEGG) in MPS II neurons. The mapping of the regulated genes in the pathway suggested a potential alteration in the calcium homeostasis in MPS II neurons. *N* = 3 clones for HC, *N* = 9 clones from 4 patients for MPS II. **C** The RNA-seq data were validated by qRT-PCR, confirming the majority of expression perturbations in several calcium signaling-related genes, as shown in **A**, in MPS II (*N* = 5 differentiations from 4 patients’ clones) neurons compared with HC (*N* = 4 differentiations from 3 clones) and ISO (*N* = 4 differentiations from 2 ISO clones from 2 patients) neurons. **D** MPS II neurons showed elevated baseline calcium levels compared to HC neurons. The intracellular calcium changes were quantified by assessing the fluorescence intensity of the Fura-2 dye at 340 nm and 380 nm wavelengths using fluorescence microscopy in 15-week HC (*N* = 4 differentiations from 2 clones), ISO (*N* = 4 differentiations from 2 ISO clones from 2 patients), and MPS II (*N* = 8 differentiations from 4 clones from 3 patients) neurons. Each dot in the histogram represents the mean of 13 time points for a single differentiated clone. **E** Thapsigargin (TG) treatment caused a greater increase in intracellular calcium levels in MPS II neurons. The differences in the fluorescence intensity ratios at 340 nm and 380 nm (340/380) after TG treatment were determined by subtracting the baseline 340/380 ratio from the post-treatment values. This difference is represented as “∆ (340/380)”. The arrow indicates the moment when TG was added. The area under the curve (AUC) of the ∆ (340/380) over a 10-min period was computed, providing a quantitative measure of changes in intracellular calcium concentration (right panel). **F** A calcium channel blocker, verapamil, rescued the altered expression of certain genes in **B** that regulate calcium signaling (*CACNA1A, P2RX3, ADRA1A*, and *PLCD3*), and the genes encoding other ion channels (*SCN9A* and *KCND2*) and a neurofilament protein (*NEFL*) in 15-week MPS II neurons (*N* = 4 differentiations from 4 patients’ clones). The data represent mean ± SEM. *p < 0.05, **p < 0.01, ***p < 0.005, analyzed by Mann–Whitney U test for **A**, **C**–**E**, and Wilcoxon signed-rank test for **F**. Cell clone details are in Supplementary Table 1.

KEGG pathway analysis identified calcium signaling as the second most influenced pathway in MPS II neurons (Fig. 3A). GSEA further confirmed this, highlighting enriched genes associated with cytosolic calcium ion concentration, positive regulation of calcium ion sequestration, and calcium channel complex in MPS II neurons (Fig. 4A, Supplementary Fig. 5A). KEGG pathway mapping (Fig. 4B) showed these enriched genes as upregulated or downregulated across calcium pathway nodes, most verified by qRT-PCR (Fig. 4C). Fura-2 calcium imaging and spectrofluorometry both indicated higher baseline intracellular calcium levels in MPS II neurons, even pre-treatment (Fig. 4D, Supplementary Fig. 5B). After Thapsigargin (TG) treatment, MPS II neurons exhibited greater calcium release than controls, suggesting disturbed endoplasmic reticulum (ER) calcium homeostasis (Fig. 4E, Supplementary Fig. 5B).

To further explore dysregulated calcium signaling, we treated 15-week HC neurons with excessive HS, a GAG accumulated in MPS II, revealing upregulation of calcium channel genes *CACNA1A* and *P2RX3* (Supplementary Fig. 5C). Conversely, GSK-3β inhibitors, CHIR99021 or Tideglusib, downregulated these genes in MPS II neurons (Supplementary Fig. 5D). Moreover, Ionomycin, a calcium ionophore, caused gene expression changes in HC neurons resembling MPS II neurons, including downregulation of *ADRA1A*, *PLCD3, MYLK2, PDE1B*, *SCN9A*, and *KCND2* (Supplementary Fig. 5E). In contrast, verapamil, a calcium channel blocker, reversed many gene expression alterations in MPS II neurons (Fig. 4F). Therefore, both HS accumulation and GSK-3β overactivity might contribute to the elevated intracellular calcium and altered calcium pathway gene expression observed in MPS II neurons. This could be rescued by a readily accessible calcium channel blocker drug.

### Selected drug candidates rescued abnormalities in MPS II neurons

**Fig. 5:**
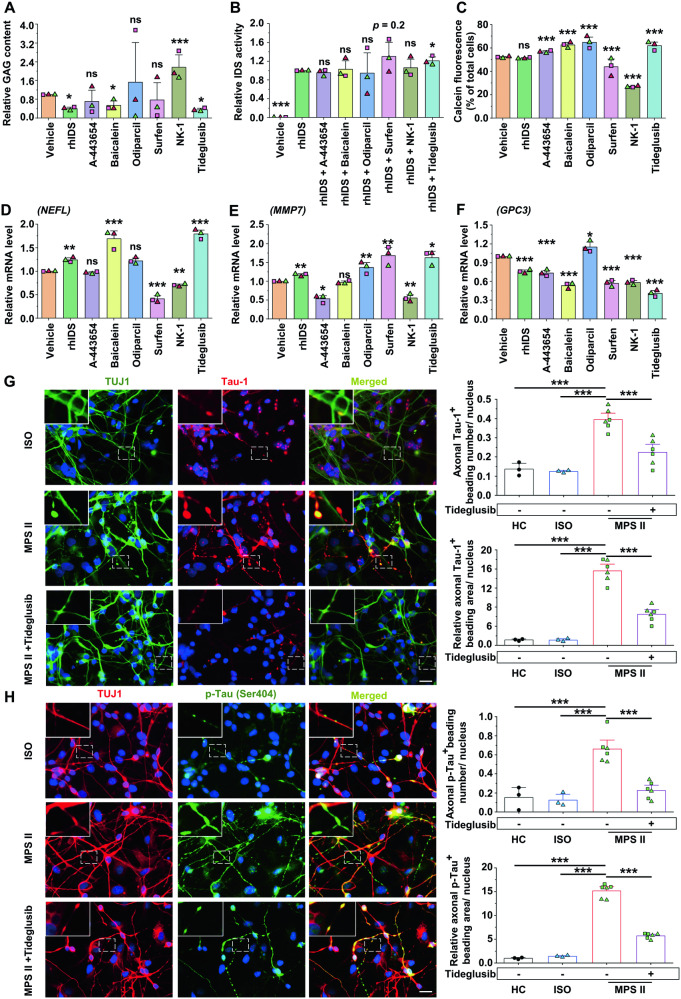
Therapeutic effects of selected drugs and chemicals on MPS II neurons. MPS II neurons were used to test the therapeutic effects of human rhIDS (20 ng/mL) and several potential drugs, including A-443654 (0.05 μM), baicalein (10 μM), Odiparcil (10 μM), surfen (10 μM), NK-1 (0.5 μM), and Tideglusib (10 μM). Solvents (vehicles) alone were used as the control. **A** GAG colorimetric assay was performed to measure the GAG content in cells that were treated with drugs for seven days. **B** IDS activity assay was performed in four-week MPS II neurons treated with rhIDS (20 ng/mL) alone or in combination with different drugs for seven days to evaluate the effects of the drugs on IDS activity. **C** The effect of drugs on the viability of MPS II neurons was assessed by flow cytometry using the live cell indicator calcein-AM after 24 h of drug treatment. The qRT-PCR showed the differential rescue effect of drugs on the mRNA levels of marker genes, such as the downregulated *NEFL* in **D**, or the DEGs of RNA sequencing, including *MMP7* in **E** and *GPC3* in **F** in the 15-week MPS II neurons treated with different drugs for 24 h. The mRNA levels were normalized to GAPDH and presented as fold change relative to vehicle-treated cells. All data were compared with the control vehicle, except in **B**, where they were compared with IDS alone, and both analyzed by Student’s *t* test. **G**, **H** IF microscopy showed that 15-week MPS II Tuj-1-positive neurons exhibited significantly increased numbers and areas of Tau-1- (**G**) and p-tau- (**H**) positive axonal beadings. Furthermore, the Tideglusib (10 μM) treatment rescued the phenotypes. Scale bar, 20 μm. Insets: magnified images of the beading regions (dotted squares). *N* = 3 clones from 3 patients for MPS II in **A**–**F**; *N* = 3 independent differentiations from 1 clone for HC and ISO, and *N* = 12 independent differentiations from 2 patients’ clones for MPS II in **G** and **H**. Data represent mean ± SEM. *p < 0.05, **p < 0.01, ***p < 0.005, analyzed by paired Student’s *t* test for **A**, **B**, **D**–**F**, chi-square for **C**, and Mann–Whitney U test for **G**, **H**. Each data point presented originates from independent differentiations. Descriptions of cell clones used are provided in Supplementary Table 1.

MPS II neurons were employed to test various potential therapeutic agents: rhIDS (MPS II drug); A-443654 (predicted by the LINCS L1000 data repository [21] to rescue the expression of *GPC3*, *MMP7*, and *NEFL*); Baicalein (Tau-1 aggregation inhibitor [22]); Tideglusib (GSK-3β inhibitor [23]); Odiparcil (GAG-clearing drug [24]); surfen (HS antagonist [25]); and NK-1 (HS/DS binder [26]). Except for rhIDS and Odiparcil, all can cross the BBB [22, 23, 25–27]. Drug dosages and durations were determined based on literature [23–28] and our cytotoxicity test (Supplementary Table 3), applied to four-week and 15-week MPS II and control neurons (Supplementary Fig. 6A). Among them, rhIDS, baicalein, and Tideglusib, significantly reduced GAGs (Fig. 5A). Only rhIDS enhanced IDS activity when administered alone (Supplementary Fig. 6B, C), with Tideglusib further boosting it in four-week (Fig. 5B), but not in 15-week neurons (Supplementary Fig. 6D). Nevertheless, combined rhIDS and Tideglusib treatment from weeks four to six, followed by rhIDS alone until week 15, improved IDS activity in MPS II neurons more than rhIDS alone (Supplementary Fig. 6E). Significant viability improvement was observed in the 15-week MPS II neurons treated with A-443654, baicalein, Odiparcil, or Tideglusib for 24 h (Fig. 5C), while rhIDS required a longer incubation (seven days) for viability enhancement (Supplementary Fig. 6F). Furthermore, qRT-PCR showed that rhIDS or Tideglusib significantly upregulated *NEFL* and *MMP7*, and downregulated *GPC3* in 15-week MPS II neurons (Fig. 5D–F). Additionally, Tideglusib decreased Tau-1 and phosphorylated tau in axon-beading regions of 15-week MPS II neurons (Fig. 5G, H). These results suggested that Tideglusib, with its multifaceted rescuing effects, was the most promising agent tested.

### Tideglusib reversed functional and morphological aberrations in MPS II neurons

**Fig. 6:**
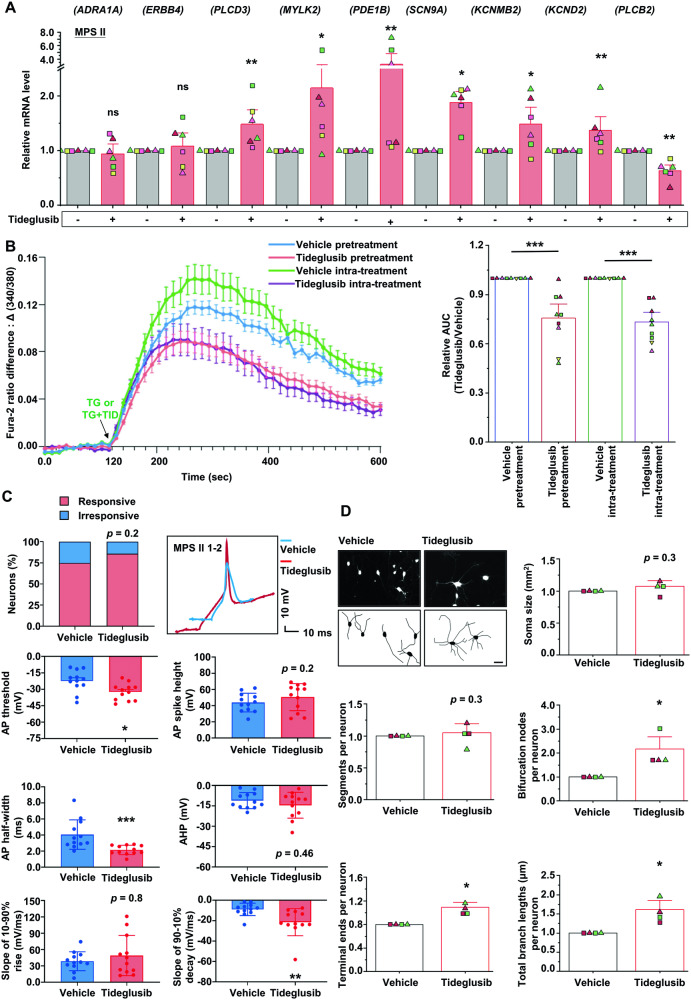
Tideglusib treatment reversed the action potential anomalies in MPS II neurons. **A** Tideglusib treatment (10 μM, 1 day) reversed the abnormal downregulation of the genes associated with calcium signaling pathways (*PLCD3, MYLK2*, and *PDE1B*) and genes encoding sodium or potassium channels (*SCN9A, KCNMB*, and *KCND2*), and reversed the abnormal upregulation of the phospholipase-encoding *PLCB2* gene in 15-week MPS II neurons (*N* = 6 clones from 4 patients). **B** Tideglusib mitigated the intracellular calcium increase caused by TG treatment in MPS II neurons. The intracellular calcium changes were quantified by assessing the fluorescence intensity of the Fura-2 dye at 340 nm and 380 nm wavelengths using fluorescence microscopy in 15-week MPS II neurons and categorized as vehicle control (*N* = 9 differentiations from 6 clones), Tideglusib pretreatment (*N* = 9 differentiations from 6 clones), and Tideglusib intra-treatment (*N* = 9 differentiations from 6 clones). To assess the effect of treatment, we subtracted the baseline 340/380 ratios from the post-treatment fluorescence intensity ratios, calculating the change, namely Δ (340/380). The Tideglusib pretreatment and Tideglusib intra-treatment groups both showed reduced ∆ (340/380) values compared to the Vehicle group. The area under the curve (AUC) of the ∆ (340/380) over a span of 10 min was computed, providing a quantitative depiction of intracellular calcium concentration changes. The arrow indicates the time when TG alone or TG combined with Tideglusib was added. Vehicle pretreatment: MPS II neurons pre-treated with the vehicle prior to TG treatment; Tideglusib pretreatment: MPS II neurons pre-treated with Tideglusib for 24 h prior to TG addition; Vehicle intra-treatment: MPS II neurons treated with TG and vehicle; Tideglusib intra-treatment: MPS II neurons treated simultaneously with both Tideglusib and TG. **C** The rescuing effect of Tideglusib on the electrophysiology of MPS II neurons. The 15-week MPS II 1-2 neurons were treated with 10 μM Tideglusib or vehicle (DMSO) for 2 weeks. The APs in MPS II neurons, as mentioned above, were measured using current-clamp recordings in response to increasing current steps (−50 to 150 pA). Tideglusib treatment trended toward an increased recordable AP percentage in MPS II neurons (left upper panel). *N* = 16 cells from 3 independent differentiations of 1 clone for vehicle group; *N* = 14 cells from 5 independent differentiations of 1 clone for Tideglusib group. *p* = 0.2, analyzed by chi-square test. Representative change of the AP morphology of MPS II neurons treated with Tideglusib compared to those treated with vehicle (right upper panel). Scale bars: 10 ms (x-axis) and 10 mV (y-axis). Histograms show the differences of AP parameters between vehicle- and Tideglusib-treated MPS II neurons, including AP threshold, AP spike height, AP half-width, after-hyperpolarization (AHP), the slope of 10–90% rise, and the slope of 90–10% decay. *N* = 12 cells from 3 independent differentiations for vehicle group; *N* = 12 cells from 5 independent differentiations for Tideglusib group. **D** The rescuing effect of Tideglusib on the neurite morphology of MPS II neurons. The 15-week MPS II neurons were treated with 10 μM Tideglusib or vehicle (DMSO) for 2 weeks. Neurite morphometrics were measured using Neurolucida Neuron Tracing software. Representative images of the 15-week MPS II neurons transduced with AAV9-*CaMKII α*-*EYFP*. Neuron morphology through fluorescence microscopy is displayed in the upper panels, while their reconstructed images by Neurolucida are shown in the lower panels. Scale bar, 10 μm. The morphometric parameters of neurites included soma size, segments, bifurcation nodes, terminal ends, and total branch lengths. *N* = 4 clones from 2 MPS II patients for each group. Data represent mean ± SEM. *p < 0.05, **p < 0.01, ***p < 0.005, analyzed by Wilcoxon signed-rank test for **A**, **B**, and **D**, Mann–Whitney U test for **C**. Cell clone details are in Supplementary Table 1.

In 15-week MPS II neurons, Tideglusib effectively corrected the aberrant expressions of the majority of aforementioned calcium pathway genes and the three AP-related DEGs: *SCN9A, KCNMB2*, and *KCND2* (Fig. 6A, Supplementary Fig. 5D).

Concordantly, Fura-2 calcium imaging demonstrated that Tideglusib significantly reduced abnormal calcium-releasing responses to TG in MPS II neurons, both in pre- and simultaneous treatments (Fig. 6B). To assess Tideglusib’s therapeutic effects on electrophysiology and neurite morphology, we established an optimal regimen for 15-week neurons (Supplementary Fig. 6G), confirming 10 µM Tideglusib’s efficacy over two weeks by activating the canonical Wnt target gene *AXIN2* [29] (Supplementary Fig. 6H), without affecting neuronal survival. Patch-clamp recordings indicated a trend of increased responsiveness in MPS II neurons and significant improvements in AP threshold, AP half-width, and 90–10% decay slope post-Tideglusib (Fig. 6C). Morphometric analysis showed that Tideglusib increased bifurcation nodes, terminal ends, and total branch lengths in MPS II neurons (Fig. 6D).

### Regulation of ion channel genes in iPSC-derived neurons by p38 MAPK and Wnt/β-catenin signaling

Finally, we investigated if decreased expression of ion channel genes (*KCND2*, *KCNMB2*, and *SCN9A*) in MPS II neurons was directly associated with dysregulated p38 MAPK or Wnt/β-catenin signaling. Treating iPSC-derived neurons with SB203580 (p38 MAPK inhibitor) or MSAB (Wnt/β-catenin inhibitor) downregulated these genes and a known common target gene (*FOSL1*) across all neuron groups (Supplementary Fig. 7A). Conversely, Anisomycin (p38 MAPK activator) or Tideglusib (Wnt/β-catenin activator) upregulated these genes, particularly *SCN9A* (Supplementary Fig. 7A). Concordantly, chromatin immunoprecipitation (ChIP) analysis confirmed β-catenin binding to the *SCN9A* promoter LEF1/TCF consensus sequence in 15-week neurons (Supplementary Fig. 7B). This suggested that dysregulated Wnt/β-catenin signaling in MPS II neurons might directly downregulate key ion channel genes, contributing to abnormal neuronal APs.

## Discussion

In the past decade, ERT has notably improved life expectancy and quality for MPS II patients, yet it falls short in addressing severe-form MPS II cases, where neuropsychiatric abnormalities persist despite ERT [5]. New approaches like intrathecal rhIDS face risks like device malfunction [4]. Izcargo® (pabinafusp alfa) exploits transcytosis for rhIDS to cross the BBB, showing initial neurocognitive improvement [30], but long-term effects are unclear. Meanwhile, alternative treatments like hematopoietic stem cell transplantation [31] and gene therapy [30], encounter efficacy debates [5] or clinical trial challenges, including complications, immunogenicity, and costs [30].

MPS II-iPSC clones have been reported in literature [32–36], and some were differentiated into neuronal populations, displaying phenotypes like GAG accumulation, increased LAMP-2 expression, apoptosis, ER and Golgi complex structural changes, and secondary lipid accumulation. However, our models, employing more mature neurons, revealed unique and previously unreported findings in MPS II neurons, including morphometric and electrophysiological abnormalities, transcriptome variations, signaling pathway dysregulation, and potential therapeutic agents. An important abnormality in MPS II neurons was axonal beading, reminiscent of axonal spheroids seen in other neurodegenerative conditions [37]. Impairment of axonal transport has been suggested as a likely cause of axonal spheroids [38]; however, further investigation is needed to ascertain its relevance in our system. Our results indicated that these abnormalities were Tau-1 positive varicosities, linked to tau aggregation. This new phenotype is reasonable since tau aggregation depends on HS [39], a GAG accumulated in MPS II. Notably, over-sulfated HS in MPS II has been hypothesized to enhance tau aggregation [40], as sulfated HS is necessary for tau’s binding to HS proteoglycans [41]. Accumulation of hyperphosphorylated tau has been reported in the brains of MPS IIIA patients [42] and MPS IIIB mice [43]. Whether tau aggregations exist in human MPS II brain pathological specimens remains to be determined, but this raises the possibility of using currently available tau-aggregation inhibitors to treat MPS II neurodegeneration, provided tau aggregations are proven to exist. Congruently, our results showed that baicalein, a tau-aggregation inhibitor, partially reversed MPS II neuron phenotypes. Notably, Tideglusib treatment also reduced axonal varicosities, indicating GSK-3β inhibition may lessen tau aggregation in MPS II, similar to its effect in AD [44]. However, in AD brains, elevated GSK-3β stems from Aβ [45], while in MPS II neurons, reduced Wnt and p38 MAPK signaling might be the cause.

The p38 MAPK signaling interacts with the Wnt/β-catenin signaling in various ways. For instance, WNT3A activates p38 MAPK through Dishevelled [46], while p38 MAPK phosphorylates GSK-3β‘s C-terminus, increasing β-catenin nuclear accumulation [47]. Additionally, p38 MAPK phosphorylates myocyte enhancer factor 2 (MEF2), enhancing MEF2’s nuclear translocation and interaction with β-catenin, activating Wnt and MEF2 target genes [48]. Moreover, AP-1, another p38 MAPK effector, synergistically activates Wnt/β-catenin target genes with β-catenin/TCF [49]. Wnt and p38 MAPK cooperate in negatively regulating autophagy. Both inhibit GSK-3β, and p38 MAPK hampers autophagy by phosphorylating ULK1 [50], a GSK-3β-activated kinase [51] that triggers autophagosome formation. Conversely, autophagy downregulates Wnt signaling by degrading Dishevelled [52] and suppresses p62-mediated p38 MAPK activation [53].

GAG accumulation in our MPS II cells was milder at the iPSC stage but worsened during neuronal differentiation, which could explain the non-significant increase in GAG levels in MPS II iPSCs compared to ISO iPSCs. Similarly, Rybová et al. [33] also found no significant increase in GAG levels in MPS II iPSCs compared to healthy controls, suggesting a physiological role of enhanced GAG synthesis during neuronal differentiation. Undegraded GAG accumulation can affect cell signaling [54]. Costa et al. reported increased Wnt signaling in *Ids* morpholino-treated zebrafish heart valves [55], and Salvalaio et al. found altered Wnt signaling in *Ids*-knockout mouse brains, with marked regional variations [56]. Our results concurred with the mixed Wnt gene regulation (both upregulation and downregulation). However, the expression of Wnt downstream genes was downregulated in our results, but unchanged in theirs [56], possibly due to differences in neuronal maturation stages and species. Reduced Wnt/β-catenin signaling led to some downstream genes’ reduced expression, which might have prompted the upregulation of specific upstream genes, compensating for the weakened signaling.

**Fig. 7:**
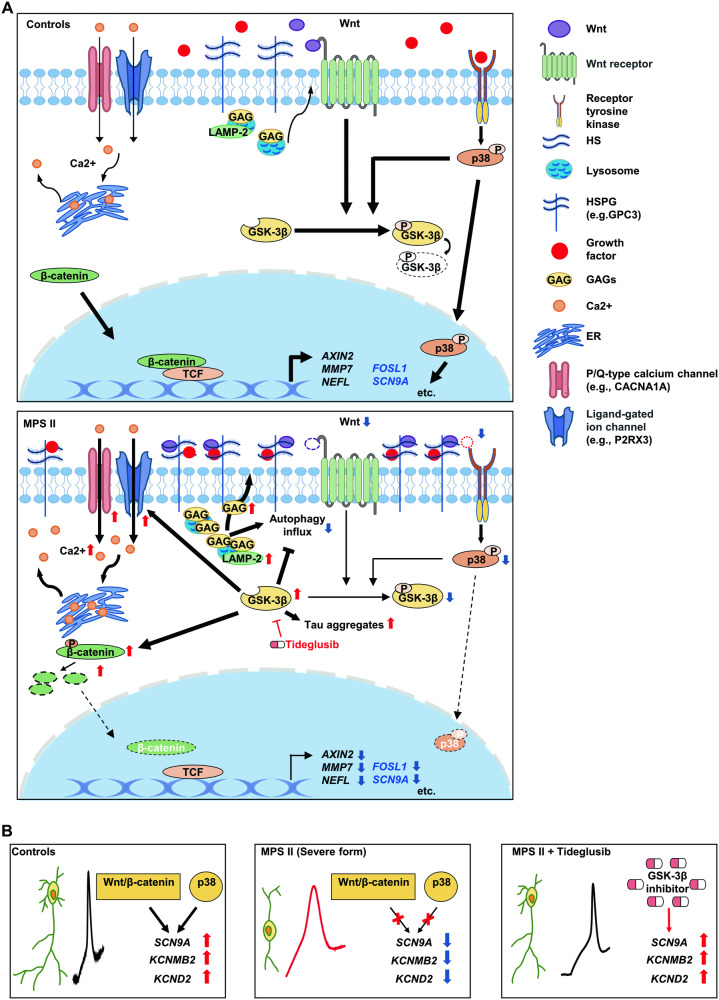
Schematic illustration of a hypothetic model for signaling pathway alterations and cellular phenotypes in MPS II neurons. **A** Defective degradation of GAGs in lysosomes leads to a decline in autophagy flux and a consequent increased accumulation of LAMP-2. Furthermore, excessive extracellular storage of GAGs results in an increased accumulation of HSPGs on the cell membrane. The excessive GAG chains on HSPGs in MPS II neurons may sequester specific ligands (such as Wnts and growth factors), thereby depressing the activation of Wnt and RTK receptors. The decrease in Wnt receptor-mediated signaling enhances GSK-3β activity. This, in turn, suppresses autophagy flux, promotes tau aggregation, and elevates intracellular calcium concentration via activation of the expression of selective genes that encode calcium channels. Additionally, it inhibits β-catenin/TCF-regulated gene expression (e.g., *AXIN2*, *MMP7*, and *NEFL*). On the other hand, reduced RTK signaling diminishes the active (phosphorylated) form of p38 MAPK and its nuclear translocation, thereby reducing both p38 MAPK-mediated inhibitory phosphorylation on GSK-3β and p38 MAPK-activated gene expression (such as *FOSL1* and genes encoding selected ion channels, e.g., *SCN9A*). The width of the arrows indicates the relative strength of regulation, and the dashed lines indicate decreased levels of nuclear translocation. Genes regulated by both p38 MAPK and β-catenin/TCF are denoted in blue font. **B** Decreased p38 MAPK and Wnt/beta-catenin signaling in MPS II neurons may lead to abnormal AP via ion channel gene dysregulation (e.g., *SCN9A*, *KCNMB2*, and *KCND2*). Tideglusib treatment restores the expression of these genes and mitigates the AP anomalies of MPS II neurons. Red arrows indicate increased expression; blue arrows indicate decreased expression.

Although the precise mechanisms of MPS II neurodegeneration remain unclear, our study proposes an intriguing model (Fig. 7) for the disease’s pathogenesis. The scenario might begin with intracellular accumulation of GAGs in lysosomes, causing lysosomal destabilization, initial autophagy induction, and subsequently autophagic buildups with increased LAMP-2 expression. Initial autophagy induction could weaken both Wnt [52, 57] and p38 MAPK [53] signaling. Extracellularly, GAGs, especially HS affected growth factor distribution and function due to their high affinity with these molecules [58]. Excessive HS-proteoglycans in MPS I and IIIB cells have been shown to sequester FGF2, reducing its signaling, which is reversible with HS-targeting proteins [59]. Jenkins et al. [60] found that Wnts’ interaction with HS in membrane proteoglycans inhibits Wnt signaling by trapping Wnt ligands. Excessive GAGs may thus sequester Wnt ligands and growth factors, decreasing their binding to frizzled receptors and receptor tyrosine kinases (RTK), reducing both Wnt and RTK-p38 MAPK signaling (Fig. 7A). This led to increased GSK-3β activity due to reduced inhibition, further suppressing Wnt/β-catenin signaling. Decreased p38 MAPK activity downregulated its downstream genes, including those shared by Wnt/β-catenin signaling (*FOSL1*) and ion channel genes (*SCN9A*, *KCNMB2*, and *KCND2*), crucial for APs (Fig. 7B) [61, 62]. Meanwhile, elevated intracellular calcium is also known to raise the AP threshold and suppress neuron excitability by activating several calcium-gated potassium currents [63]. Additionally, it also contributes to neuronal death [64].

Dysregulated calcium homeostasis in MPS II neurons in calcium imaging aligned with calcium pathway gene alterations in KEGG pathway mapping. Upregulated genes like *CACNA1A*, *P2RX3*, and *PLCB2* might increase baseline cytosolic calcium and ER calcium release upon TG treatment in calcium imaging. Lysosomal storage diseases, like Gaucher disease [65] and Niemann-Pick type C1 [66], demonstrate how accumulated substrates can disrupt calcium homeostasis by affecting ryanodine receptors and calcium ATPases/Na^+^/Ca2^+^ exchangers, respectively.

Likewise, accumulated GAGs in MPS II neurons might influence calcium channels or calcium-handling proteins. This was supported by our qRT-PCR results, showing upregulation of *CACNA1A* and *P2RX3* after excessive HS treatment, linking HS accumulation to calcium homeostasis changes. Moreover, increased GSK-3β activity might also contribute. GSK-3β is known to interact with inositol trisphosphate receptor, influencing its phosphorylation and calcium release from ER [67]. GSK-3β overexpression in mice correlates with reduced *Serca2a* expression and elevated cytosolic calcium in cardiomyocytes [68]. Thus, elevated GSK-3β in MPS II neurons likely altered calcium homeostasis. Our qRT-PCR supported this, showing that GSK-3β inhibitors downregulated *CACNA1A* and *P2RX3*.

Other gene expression changes in the KEGG calcium pathway might be secondary or compensatory. Elevated intracellular calcium may upregulate MYLK2 [69] and PDE1B [70] enzyme activities, causing their mRNA compensatory downregulation. Supporting this, Ionomycin-induced calcium elevation downregulated *MYLK2* and *PDE1B* in control neurons. Additionally, *PLCD3* downregulation is a secondary effect of increased intracellular calcium [71], as supported by Ionomycin’s effect on this gene.

Our results revealed Tideglusib’s effectiveness in correcting morphological and electrophysiological aberrations of mature MPS II neurons. Tideglusib, a selective non-ATP-competitive GSK-3β inhibitor [72], has been applied in clinical trials for several neurological diseases, including AD [73], myotonic dystrophy type 1 [74], progressive supranuclear palsy [75], and amyotrophic lateral sclerosis [76]. This suggests the involvement of GSK-3β in a fundamental pathway shared among multiple neurodegenerative disorders. However, its definite efficacy and optimal dosing require further validation in larger clinical trials. Given its ability to penetrate the BBB [23] and its established human pharmacokinetics and safety profile [77], Tideglusib holds significant potential for treating MPS II neurodegeneration, either alone or in combination with other therapies, pending validation through animal studies.

This study unveils novel morphological and functional abnormalities in mature MPS II neurons, and their mechanistic association with dysregulated signaling pathways. It highlights Tideglusib, a GSK-3β inhibitor, as a promising candidate for correcting neuronal phenotypes, offering valuable insights for MPS II therapy. These discoveries advance our comprehension of MPS II neurodegeneration and suggest new therapeutic strategies.

## Materials and methods

### Human subjects and sample collection

Blood was collected from four male patients with the severe form of MPS II diagnosed at the MacKay Memorial Hospital in Taiwan based on their clinical features, low IDS enzyme activities, and pathogenic mutations of the *IDS* gene, with their parents’ written informed consent. The protocol was approved by the Research Ethics Committee of both the National Taiwan University Hospital and MacKay Memorial Hospital. The ages and *IDS* mutations of these patients were as follows: Patient 1, 29 years, CGG > TGG (R468W) point mutation; Patient 2, 14 years, 1214_1215 del CT; Patient 3, 20 years, IVS7+5G>C; Patient 4, 28 years, c.1122C>T, (p.G374G). The collected blood in heparin-containing tubes was mixed with Histopaque (Sigma-Aldrich, MA, USA) at a 1:1 ratio, and the PBMCs were isolated by centrifugation at 400 × *g* for 30 min at room temperature.

### Generation of MPS II-iPSC

The iPSCs were derived using a nonintegrated Sendai virus system (CytoTune™-iPS 2.0 Sendai Reprogramming Kit, Thermo Fisher Scientific, MA, USA) according to the manufacturer’s instructions. Briefly, MPS II-PBMCs were plated at a density of 1 × 10^6^ cells/well in four well plates in StemPro®-34 SFM medium (Thermo Fisher Scientific, MA, USA) supplemented with cytokines (100 ng/mL stem cell factor, 100 ng/mL FMS-like tyrosine kinase 3, 20 ng/mL IL-3, 20 ng/mL IL-6) and Glutamax (Thermo Fisher Scientific, MA, USA). Half of the culture medium was replaced each day for four days, and a combination of Sendai virus expressing human OCT3/4, SOX2, KLF4, and MYC was added to the target cells. Three days later, the infected PBMCs were replated onto mitomycin-C-inactivated MEFs. Two days later, the StemPro®-34 SFM medium (Thermo Fisher Scientific, MA, USA) without cytokines was changed for two days and then switched to StemFlex (Thermo Fisher Scientific, MA, USA) medium. The iPSC clones grown for three weeks were then selected, expanded, and subjected to further characterization, as described below. Finally, three optimal iPSC clones that fulfilled the criteria for human iPSCs [78] were selected for each patient and labeled as -1, -2, and -3.

### Healthy male control iPSCs and the culture of iPSCs

Healthy male control iPSC clones (HC-1~3), which were obtained from the Bioresources Collection and Research Center (BCRC), Taiwan, with the original names of IBMS-iPSC-02-07 (SC81031), TVGH-iPSC-02-07 (SC81033), and NTUH-iPSC-02-02 (SC81035), respectively, were reprogrammed by Sendai virus as human iPSC clones from three male subjects, generated by the Human Disease Induced Pluripotent Stem Cell Service Consortium (National Core Facility for Biopharmaceuticals, Taiwan). All iPSCs were cultured on Matrigel (Corning, NY, USA) with StemFlex (Thermo Fisher Scientific, MA, USA) at 37 °C in a humidified atmosphere containing 5% CO_2_.

### In vitro differentiation of MPS II-iPSCs

For in vitro differentiation, MPS II-iPSC colonies were dispersed into small clumps using dispase (Thermo Fisher Scientific, MA, USA; 2 mg/ml for 10 min) and transferred onto ultralow attachment plates (Corning, NY, USA) for embryoid body (EB) formation in iPSC medium, which was changed every day for four days. The EBs were then transferred to culture dishes coated with 0.1% gelatin and fetal bovine serum (FBS)-containing medium. During the 14-day differentiation period, the medium was changed every two days.

### Teratoma formation assay

Approximately 1–1.5 × 10^7^ MPS II-iPSC cells were injected subcutaneously into the backs of 6–8-week-old BALB/c nude mice (National Laboratory Animal Center, Taipei, Taiwan). Teratomas were allowed to develop for up to 8–10 weeks and were then excised and fixed with 4% paraformaldehyde overnight at 4 °C before embedding in paraffin for sectioning. The samples were cut to 7 µm in thickness and stained with hematoxylin and eosin stain (H&E) for histological analysis. All animal experiments were undertaken according to the Guidebook for the Care and Use of Laboratory Animals (published by the Chinese-Taipei Society of Laboratory Animal Sciences) and approved by the Institutional Animal Care and Use Committee at National Taiwan University, College of Medicine. The well-being of the animals was monitored daily, and the animals had free access to food and water.

### Karyotyping

To perform karyotyping on the iPSC clones, we first arrested the cell cycle of iPSCs by treating them with 10 μg/mL of colcemid (Thermo Fisher Scientific, MA, USA) for 20 min at 37 °C. We then incubated the cells with 75 mM hypotonic KCl, which led to nuclear swelling. Next, the cells were fixed using a 3:1 methanol/glacial acetic acid solution. Metaphase chromosomes were collected and subjected to Giemsa staining to analyze the G-bands. We counted at least 20 metaphase spreads for cytogenetic analysis.

### Reverse transcription-polymerase chain reaction (RT-PCR) and quantitative real-time reverse transcription PCR (qRT-PCR)

In brief, total RNA was isolated and purified with the GENEzol TriRNA Pure Kit with DNase I treatment (Geneaid, Taiwan), and the cDNAs were synthesized with the SuperScript™ III Reverse Transcriptase (Thermo Fisher Scientific, MA, USA) according to the manufacturer’s instructions. RT-PCR was conducted using 2X SuperRed PCR Master Mix (BIOTOOLS Co., Ltd, Taiwan) according to the manufacturer’s instructions. A 20 μL solution containing 5 ng total cDNA was used for PCR in the following conditions: 95 °C 3 min; 35 cycles of 95 °C 30 s; 50–60 °C 30 s; and 72 °C 1–5 min. They were kept at 4 °C until electrophoresis using 1–2% agarose gel (depending on the size of the band). qRT-PCR was performed in 25 μL of reaction solution containing 5 ng RNA equivalent cDNA template, 400 nM of each primer, and 1X SYBR Green (Thermo Fisher Scientific, MA, USA). The PCR reactions were set up using a QuantStudio™ 5 Real-Time PCR System (Thermo Fisher Scientific, MA, USA). RNA levels were normalized to the GAPDH level and calculated as the delta-delta threshold cycle (ΔΔCT). Four replicate datasets were analyzed for each gene.

### Alkaline phosphatase staining

Alkaline phosphatase staining was performed using the VECTOR Blue Alkaline Phosphatase Substrate kit (Vector Laboratories, CA, USA) according to the manufacturer’s instructions. Microscopic images were acquired by ZEISS Axio Observer 7 (Carl ZEISS, Jena, Germany).

### IF staining

The samples were fixed in 4% paraformaldehyde for 20 min at room temperature, permeabilized with 0.1% Triton X-100 (5 min), and blocked in an FBS-BSA blocking buffer for 1 h at room temperature and incubated with the primary antibodies overnight at 4 °C. We also used Alexa 488/594-conjugated secondary antibodies (Biolegend, CA, USA). DAPI was used to stain the nuclei. The fluorescence microscopic images were acquired using ZEISS Axio Observer 7 (Carl ZEISS, Jena, Germany) and analyzed using ImageJ software (Fiji edition, National Institutes of Health, USA).

### Generation of *IDS* mutation-corrected isogenic control (ISO) iPSCs

Base editing of the mutated *IDS* gene in MPS II-iPSCs derived from Patient 1 and Patient 4 was performed by transfecting cells (Pt1-4 and Pt 4-15 clones) with ABE8e-NG HiFi vectors (Gene Knockout/In Cell Line Modeling Core at the National Taiwan University College of Medicine, Taiwan), which contained Cas9 H840 nickase and sgRNA sequences specific to exon 8 or exon 9 of the *IDS* gene. The transfection was done using the transIT-LT1 reagent (Mirus Bio, WI, USA) according to the manufacturer’s instructions. Single-cell clones (corrected clones) were obtained by antibiotic selection and validated by Sanger sequencing and Edit R [79].

### Neuronal differentiation of iPSCs

The neuronal differentiation of iPSCs was performed as previously described [80]. Briefly, iPSCs were dissociated using Accutase (FoliBio, Taiwan) and seeded on Matrigel-coated plates supplemented with StemFlex medium. The cells were then cultured in 3 N medium (DMEM/F12, neurobasal medium, non-essential amino acids, sodium pyruvate, Glutamax; penicillin-streptomycin, B27, N2, 2-mercaptoethanol, all from Thermo Fisher Scientific, MA, USA) containing 10 μM SB431542 (Selleckchem, TX, USA) and 1 μM Dorsomorphin (Cayman Chemical, MI, USA) for 12 days. The formed neuroepithelial clumps were digested by dispase, reseeded on Matrigel-coated plates, and incubated in 3 N medium containing 20 ng/ml bFGF (Thermo Fisher Scientific, MA, USA) for four days. Next, the cells were maintained in a 3 N medium without bFGF, which was replaced every three days until used for the experiments. After four weeks of differentiation, TUJ1 expression was detected by flow cytometry (FACS Calibur, BD Biosciences, NJ, USA). Neuronal differentiation for more than four weeks was also performed using a 3 N medium without bFGF.

### IDS activity determination

IDS activity was determined as previously described [81]. Briefly, iPSCs and the derived neurons were used to measure the activity of IDS enzymes. Homogenates were made in water by sonication in a Bioruptor Plus sonication device (Diagenode, NJ, USA). The protein concentration was determined using a BCA protein assay kit (Merck-Millipore, Darmstadt, Germany). To measure IDS activity 10 μL (10 μg) of proteins were first incubated with 10 μL of 4-methylumbelliferyl-α-iduronate 2-sulfate (Moscerdam Substrates, Oegsteest, Netherlands) at 37 °C for 4 h. Second, the reaction was added with 20 μL McIlvains’ phosphate/citric acid buffer and 5 μL lysosomal enzymes purified from bovine testis (LEBT-M2, Moscerdam Substrates, Oegsteest, Netherlands) solution and incubated at 37 °C for 24 h. Stop buffer (0.5 M NaHCO_3_/0.5 M Na_2_CO_3_ buffer, pH 10.7 + 0.025% Triton X-100) was then added to terminate the reaction, and the fluorescence of 4-methylumbelliferyl-α-iduronate 2-sulfate was measured using a SpectraMax i3x Multi-Mode Microplate reader (Molecular Devices, CA, USA).

### XTT assay

The 4-week and 15-week control and MPS II neurons were seeded in 96-well Matrigel-coated plates and incubated with XTT labeling mixture (Cayman Chemical, MI, USA). After 2 h, the supernatant was pipetted into 96-well plates, and the conversion of XTT to formazan was quantified by measuring the spectral absorbance at 450 nm using a SpectraMax i3x Multi-Mode Microplate reader (Molecular Devices, CA, USA).

### Cellular glycosaminoglycan content

Sulfated proteoglycans and GAGs were quantified using the 1,9-dimethylmethylene blue (DMMB; Sigma-Aldrich, MA, USA) protocol [82]. The iPSCs and neurons were digested in a prepared papain solution (containing 1 mM EDTA, 2 mM DTT, and 0.3 mg/mL papain enzyme) (Sigma-Aldrich, MA, USA) at 60 °C for 1 h. Iodoacetic acid (Sigma-Aldrich, MA, USA) was then added to a final concentration of 10 mM, followed by the addition of 0.5 ml of 50 mM Tris/HCl (Sigma-Aldrich, MA, USA). Briefly, 20 μL of sample was mixed with 200 μL of DMMB reagent, and absorbance was read at 525 nm. A standard curve was established from chondroitin-6-sulfate (Sigma-Aldrich, MA, USA) to compare absorbance for the samples. The DNA content was measured using a fluorometric assay. Total GAG levels were normalized to total DNA content. Aliquots of the sample digestion were stained with 200 μL of Hoechst33258 (Cayman Chemical, MI, USA) working solution (2 μg/mL). The fluorescence intensities were then detected at 355 nm for excitation and 460 nm for emission. Both Hoechst-stained DNA and GAG contents were measured by a SpectraMax i3x Multi-Mode Microplate reader (Molecular Devices, CA, USA).

### Electrophysiology and neurite length detection

AAV8-*hSyn-EGFP*, AAV9-*CaMKII α*-*EYFP*, and AAV9-*mDlx-GFP* (titer: ~3 × 10^13^ genome copies per milliliter; Addgene, MA, USA) were used to label iPSC-derived neurons (14 weeks of differentiation) used in the electrophysiology study. Whole-cell patch-clamp recordings were performed when the neurons were differentiated for 16–17 weeks. The coverslips containing neurons were transferred to an immersion-type recording chamber mounted on an upright microscope (Axio Examiner D1, Carl ZEISS, Jena, Germany) and continuously perfused with oxygenated artificial cerebrospinal fluid consisting of 124 mM NaCl, 3 mM KCl, 2 mM CaCl_2_, 1 mM MgCl_2_, 1.25 mM NaH_2_PO_4_, 26 mM NaHCO_3_ and 20 mM glucose (pH 7.4, 295 mOsmol) at a rate of 2–3 mL/min and maintained at 30–32 °C. Neurons were viewed using Nomarski optics. Patch-clamp recordings of iPSC-derived neurons were performed using patch pipettes pulled from borosilicate glass tubing (1.5-mm outer diameter, 0.32-mm wall thickness; Sutter, Novato, CA, USA), with a resistance of approximately 6–8 MΩ when filled with the internal solution containing 100 mM K-gluconate, 20 mM KCl, 0.2 mM EGTA, 10 mM HEPES, 4 mM ATP-Mg, 0.3 mM GTP-Na, and 0.05% neurobiotin (pH 7.2, 295 mOsmol) (NEUROBIOTIN Tracer, SP-1120, Vector Laboratories, Inc., Burlingame, CA). Recordings were performed in current-clamp mode using a patch amplifier (Multiclamp 700 B; Molecular Devices, CA, USA).

The internal solution contained 100 mM K-gluconate, 20 mM KCl, 0.2 mM EGTA, 10 mM HEPES, 4 mM ATP-Mg, 0.3 mM GTP-Na, and 0.05% neurobiotin (pH 7.2, 295 mOsmol). The bridge was balanced, and the current was applied to the neurons to reach and maintain a nominal membrane potential of −70 mV. Neurons were accepted for further analysis only if the action potential (AP) exceeded 0 mV. The AP threshold was determined when dVm/dt reached 10 V/s. The properties of a single AP were monitored from the first AP elicited by the step depolarizing protocol: intracellular current was injected from −50 to 180 in 10 pA steps. The AP spike height was measured from the threshold to the peak of the spike. After the AP spike, after-hyperpolarization (AHP) was determined as the voltage difference between the threshold and the negative voltage point. The half-width of the AP was measured as the width at a half-maximal spike. The 10–90% rising slope and 90–10% decay slope of AP were determined using Clampfit 10.6 software (Molecular Devices, CA, USA). For accurate neuron reconstruction directly from an automatically inverted fluorescence microscope, images were acquired by ZEISS Axiovert 200 M (Carl ZEISS, Jena, Germany) and analyzed using Neurolucida software (MBF Bioscience, VT, USA).

### RNA sequencing and gene pathway analysis

The extracted total RNAs from the 15-week HC neurons (three clones) and MPS II neurons (nine clones: three clones derived from Patient 1 and two clones derived from each of Patients 2, 3, and 4) were subjected to quality control, RNA sequencing, and analyses using the BIOTOOLS (BIOTOOLS Co., Ltd, Taiwan) platform. Briefly, Trimmomatic software was used to filter and remove low-quality reads from the raw data, resulting in clean reads, which were then mapped to the GRCh38 reference genome using HISAT2. The featureCounts software was used to count the number of raw reads per gene. The read count matrix was normalized by methods that included Relative Log Expression, Trimmed Mean of M-values, and Fragments Per Kilobase of transcript per Million mapped reads to correct for differences in sequencing depth and gene length. Differential gene expression analysis was performed using either DESeq2 or DEGseq/edgeR. Genes with a |Fold Change| > 2 and corrected *p* value < 0.005 were considered differentially expressed. The Kyoto Encyclopedia of Genes and Genomes (KEGG), ingenuity pathway analysis (IPA), and gene set enrichment analysis (GESA) were used to analyze functional enrichment. The RNA-seq dataset in this study was deposited in the Gene Omnibus Database (GSE202344).

### Western blotting

For total protein extraction, the cultured iPSC-derived neurons were lysed with RIPA buffer (50 mM Tris, 150 mM NaCl, 100 mM EDTA, 1% sodium deoxycholate, 0.1% Triton X-100, 0.1% SDS, pH 7.4) at −80 °C for 30 min and centrifuged at 13,000 × *g* for 5 min (4 °C). The cell lysates were then collected.

For the extraction of cytoplasmic and nuclear fractions, cultured iPSC-derived neurons were first lysed in cytoplasmic fraction buffer (10 mM Tris, 10 mM KCl, 1 mM MgCl2, 0.1% Triton X-100, pH 7.4) on ice for 30 min and centrifuged at 13,000 × *g* for at 4 °C for 5 min, and the cytoplasmic fractions were collected. The pellet was then washed with cytoplasmic fraction buffer and centrifuged at 100 × *g* at 4 °C for 10 min three times to remove the residual cytoplasmic fraction. The pellet was then lysed in radioimmunoprecipitation assay buffer, frozen at −80 °C for 30 min, and centrifuged at 13,000 × *g* at 4 °C for 5 min. The supernatants were then collected as nuclear fractions. Protein concentrations in the cell lysates were determined using a BCA Protein Assay Kit (Merck-Millipore, Darmstadt, Germany). Cell lysates containing 30 μg of protein per sample were electrophoresed on a sodium dodecyl sulfate-polyacrylamide gel and transferred to a polyvinylidene difluoride membrane. The membrane was incubated with primary antibodies at 4 °C overnight, followed by washing with Tris-buffered saline with 0.1% Tween 20 three times and incubation with horseradish peroxidase-conjugated appropriate secondary antibodies for 1 h at room temperature. The proteins were detected using an ECL Western blotting reagent (RPN2235; Cytiva, MA, USA) with an image analyzer (FUSION Solo S, Vilber, Paris, France). The intensities of the bands were quantified using ImageJ (National Institutes of Health, USA).

### Flow cytometry analysis and fluorescence-activated cell sorting (FACS) sorting

Cells were fixed with 4% paraformaldehyde for 20 min at room temperature. Depending on the antibody used, cells were permeabilized with either 0.1% Triton X-100 (for most antibodies) or 0.1% saponin (specifically for LC3B staining) for 5 min. After blocking in FBS-BSA buffer for 1 h at room temperature, cells were incubated with primary antibodies overnight at 4 °C. This was followed by an incubation with Alexa 488/594-conjugated secondary antibodies (Biolegend, CA, USA). Flow cytometry analyses were conducted on a FACSCalibur cytometer (BD Biosciences, NJ, USA), with data acquisition using CellQuest software. For cell sorting, a FACSAria IIIu instrument (BD Biosciences, NJ, USA) was utilized. In all cases, a minimum of 10,000 cells per condition were assessed, using linear gains for light scatter channels and logarithmic gains for fluorescence channels. Data were analyzed using FlowJo software (BD Biosciences, NJ, USA).

### Luciferase reporter assay

The iPSC-derived 15-week neurons were infected with the pLAS5w-GFP-Puro-TCF virus. GFP-positive cells with equal mean fluorescent intensity were sorted and seeded on a 96-well plate by FACSAria III (BD Biosciences, NJ, USA), and luciferase activity was analyzed using a luciferase assay kit (Promega Luciferase Assay System, Promega, WI, USA).

### Assays for cell viability

Membrane-permeable dyes calcein acetoxymethyl ester (calcein-AM; Biolegend, CA, USA) was prepared as stock solutions of 1 mM stored at −20 °C. The cells were divided into two tubes and with or without treated with calcein-AM (1 μM, 20 min) at 37 °C to assay the degrees of viability, respectively. The results were analyzed by flow cytometry, as described above.

### ChIP analysis

The 17-week-old MPS II neurons were first crosslinked with 1% formaldehyde solution, followed by reverse crosslinking with 0.125 M glycine solution. The cells were then lysed on ice for 15 min in ChIP lysis buffer and sonicated for 30 sec on and 30 sec off in five cycles using a Bioruptor Plus sonication device (Diagenode, NJ, USA). The isolated 200–1000 base pairs of DNA fragments were immunoprecipitated with anti-non-phosphorylated β-catenin antibody (Cell Signaling, MA, USA) at 4 °C overnight, followed by incubation with 50 µl of protein G (Cytiva, MA, USA) at 4 °C for 4 h. The recovery of DNA fragments was achieved by reversing the crosslinking process using 5 mM NaCl and 40 µg RNaseA at 65 °C overnight, followed by 4 µl (10 mg/ml) of proteinase K (Qiagen Inc., CA, USA) at 60 °C for 1 h. The immunocomplex was purified by ethanol precipitation after being washed and eluted from beads. The human SCN9A promoter region was detected using PCR and quantified using qRT-PCR.

### Intracellular calcium measurements

The intracellular calcium levels in neurons were measured either by calcium imaging or microplate reader method. The calcium imaging was performed utilizing a Nikon Ti microscope equipped with a Qi2 camera and a CFI Plan Apochromat Lambda D objective lens (Nikon, Japan). Alternatively, the fluorescence from neurons in flat-bottom black microplates (Thermo Fisher Scientific) was detected using a SpectraMax i3x Multi-Mode Microplate reader (Molecular Devices, CA, USA). To start the experiment, neurons (3.5 × 10^4^ cells/well) were cultured in 96-well Clear (for calcium imaging) or Black (for microplate reader method) Bottom Plates (Thermo Fisher Scientific) and were loaded with 4 µM Fura-2, 0.08% Pluronic™ F-127, and 1 mM probenecid (all from Thermo Fisher Scientific) in extracellular buffer (ECB), which includes 125 mM NaCl, 5 mM KCl, 1.5 mM MgCl_2_, 20 mM HEPES, 10 mM Glucose, and 1.5 mM CaCl_2_, for 30 min at room temperature. The dye was then washed out in ECB, and cells were incubated for an additional 30 min to allow for complete de-esterification of the dye. Baseline intracellular calcium levels were measured at 340 nm and 380 nm wavelengths either by microscopy or a microplate reader over a two-min period. In microscopy, the mean calcium fluorescence levels were quantified from 50 neurons per time point with ImageJ software. A minimum of four wells were utilized for biological replicates. Subsequently, neurons were treated with 4 µM Thapsigargin and 3 mM EGTA (both from Thermo Fisher Scientific) to induce immediate calcium shifts. Post-treatment images were captured at both 340 nm and 380 nm wavelengths over a 9-min period, while the post-treatment fluorescence was alternatively measured at the same wavelengths using a microplate reader over a 15-min period. The fluorescence intensity ratios at 340 nm and 380 nm (F340/F380) were then obtained from both methods. The differences between post-treatment and baseline F340/F380 ratios, represented as “delta F340/F380”, were calculated. The integrals of the delta F340/F380 ratio plotted against time were computed to illustrate changes in intracellular calcium levels using GraphPad Prism 8 Software (GraphPad Software, CA, USA).

### Statistical analysis

All data were presented as mean plus standard error of the mean (SEM), with the number of biological replicates (n) shown in figure legends. The Mann–Whitney U test, unpaired or paired Student’s *t* test, Wilcoxon signed-rank test, Welch’s *t* test, and chi-square test were performed using GraphPad Prism 8 Software (GraphPad Software). *P* values are reported as follows: ns, nonsignificant; *p < 0.05; **p < 0.01; ***p < 0.005.

### Supplementary information

supplementary Figures and Legends

original full-length Western blots

supplementary material

## Data Availability

The RNA sequencing data in this study were deposited in the Gene Expression Omnibus (GEO): GSE202344. Please visit and enter the token “azmvwuaglpetlgd”.
