# Supplementary material for: Mature neurons from iPSCs unveil neurodegeneration-related pathways in mucopolysaccharidosis type II: GSK-3β inhibition for therapeutic potential
Source: Cell Death Dis. 2024 Apr 29;15(4):302. doi: 10.1038/s41419-024-06692-9 (PMC11058230; doi:10.1038/s41419-024-06692-9)
Supplement: Supplementary file 1 — supplementary Figures and Legends [file 41419_2024_6692_MOESM1_ESM.pdf]

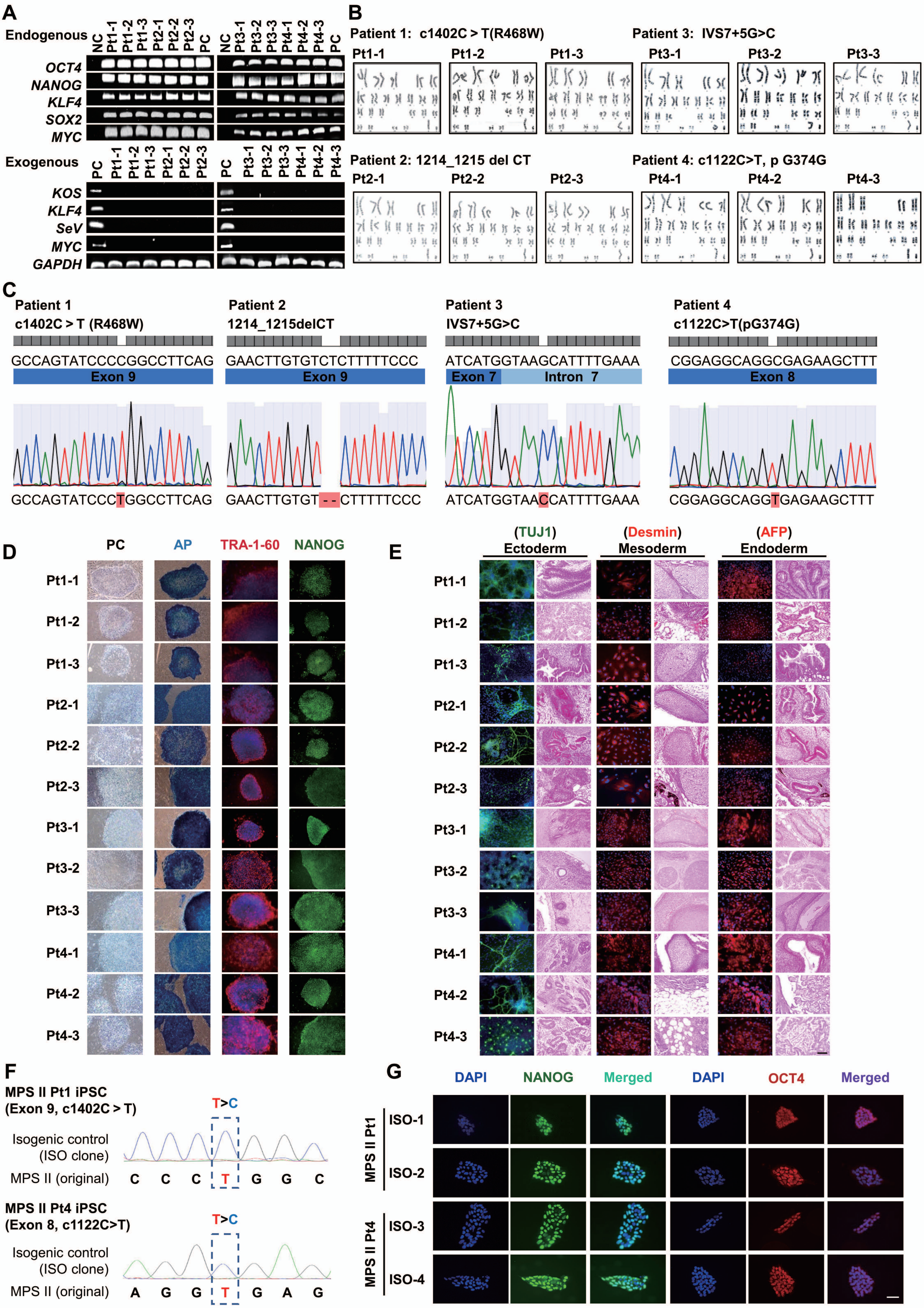
A
Endogenous
NC Pt1-1 Pt1-2 Pt1-3 Pt2-1 Pt2-2 Pt2-3 PC
NC Pt3-1 Pt3-2 Pt3-3 Pt4-1 Pt4-2 Pt4-3 PC
OCT4
NANOG
KLF4
SOX2
MYC
Exogenous
PC Pt1-1 Pt1-2 Pt1-3 Pt2-1 Pt2-2 Pt2-3
PC Pt3-1 Pt3-2 Pt3-3 Pt4-1 Pt4-2 Pt4-3
KOS
KLF4
SeV
MYC
GAPDH
B
Patient 1: c1402C > T(R468W)
Pt1-1 Pt1-2 Pt1-3
Patient 3: IVS7+5G>C
Pt3-1 Pt3-2 Pt3-3
Patient 2: 1214_1215 del CT
Pt2-1 Pt2-2 Pt2-3
Patient 4: c1122C>T, p G374G
Pt4-1 Pt4-2 Pt4-3
C
Patient 1
c1402C > T (R468W)
GCCAGTATCCCCGGCCTTCAG
Exon 9
GCCAGTATCCCTGGCCTTCAG
Patient 2
1214_1215delCT
GAACTTGTGTCTCTTTTTCCC
Exon 9
GAACTTGTGT - - CTTTTTCCC
Patient 3
IVS7+5G>C
ATCATGGTAAGCATTTTGAAA
Exon 7
Intron 7
ATCATGGTAACCATTTTGAAA
Patient 4
c1122C>T(pG374G)
CGGAGGCAGGCGAGAAGCTTT
Exon 8
CGGAGGCAGGTGAGAAGCTTT
D
PC AP TRA-1-60 NANOG
Pt1-1 Pt1-2 Pt1-3 Pt2-1 Pt2-2 Pt2-3 Pt3-1 Pt3-2 Pt3-3 Pt4-1 Pt4-2 Pt4-3
E
(TUJ1) Ectoderm
(Desmin) Mesoderm
(AFP) Endoderm
Pt1-1 Pt1-2 Pt1-3 Pt2-1 Pt2-2 Pt2-3 Pt3-1 Pt3-2 Pt3-3 Pt4-1 Pt4-2 Pt4-3
F
MPS II Pt1 iPSC
(Exon 9, c1402C > T)
T>C
Isogenic control
(ISO clone)
MPS II (original)
C C C T G G C
MPS II Pt4 iPSC
(Exon 8, c1122C>T)
T>C
Isogenic control
(ISO clone)
MPS II (original)
A G G T G A G
G
DAPI NANOG Merged DAPI OCT4 Merged
MPS II Pt1
ISO-1
ISO-2
MPS II Pt4
ISO-3
ISO-4

Supplementary Figure 1. Generation and characterization of the MPS II-specific and isogenic control iPSCs. Related to Figure 1. A PCR analysis demonstrated the expression of endogenous pluripotency markers and the silencing of Sendai viral genes in MPS II-iPSC clones. Positive control (PC): cDNA from HC-2 (TVGH-02-07) for endogenous genes and Sendai viral cDNA from PBMCs for exogenous genes. Negative control (NC): MPS II PBMC cDNA pre-Sendai virus infection. **B** Chromosomal studies confirmed normal male karyotypes in all MPS II-iPSC clones. **C** DNA sequencing revealed specific *IDS* mutations in MPS II-iPSCs, indicated by brick-red boxes. **D** Representative phase contrast (PC), alkaline phosphatase (AP), and immunofluorescence (IF) images of pluripotency markers TRA-1-60 and NANOG in all MPS II-iPSCs. DAPI counterstain (blue). Scale bar: 200 µm. **E** Differentiation of MPS II-iPSCs into three germ layers, both in vitro and in vivo, is illustrated via IF staining and teratoma formation, respectively. DAPI counterstain. AFP: alpha-fetoprotein. **F** Isogenic control (ISO) iPSCs were generated from MPS II Pt1-2 and MPS II Pt4-2 cell lines using an A-to-G base editing system (ABE8e-NG HiFi vectors). Validated single-cell clones were isolated post-antibiotic selection. **G** Expression of pluripotency markers (NANOG and OCT4) in ISO clones, derived from MPS II-iPSC 1-2 and MPS II-iPSC 4-2 lines, is demonstrated in IF images. DAPI counterstain (blue); Scale bar: 50 µm. Cell lines and clone details are in Supplementary Table 1.

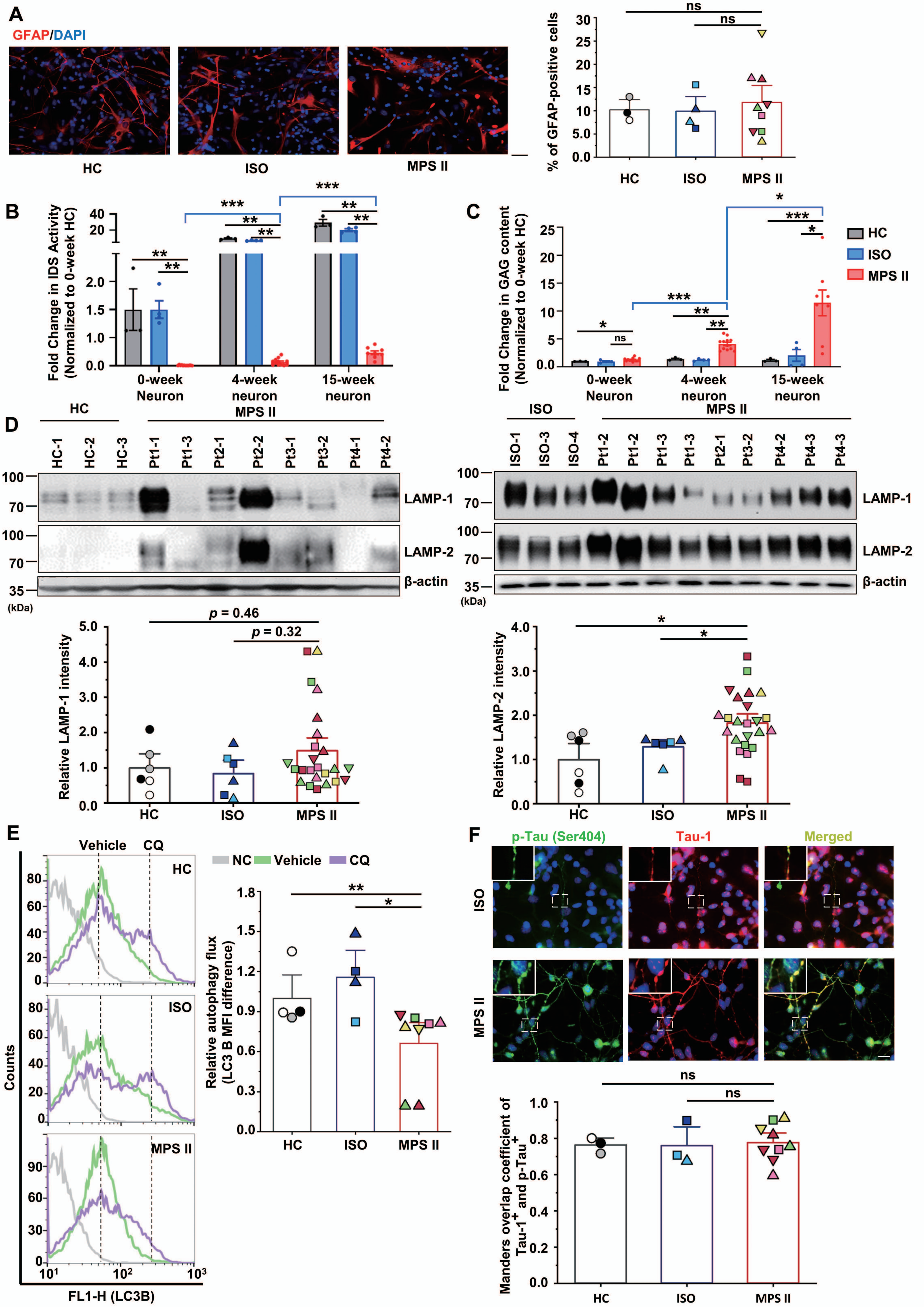
A
GFAP/DAPI
HC
ISO
MPS II
% of GFAP-positive cells
ns
B
Fold Change in IDS Activity (Normalized to 0-week HC)
0-week Neuron
4-week neuron
15-week neuron
C
Fold Change in GAG content (Normalized to 0-week HC)
HC
ISO
MPS II
D
HC-1
HC-2
HC-3
Pt1-1
Pt1-3
Pt2-1
Pt2-2
Pt3-1
Pt3-2
Pt4-1
Pt4-2
ISO-1
ISO-3
ISO-4
Pt1-2
Pt1-3
Pt2-1
Pt3-2
Pt4-2
Pt4-3
LAMP-1
LAMP-2
β-actin
(kDa)
Relative LAMP-1 intensity
p = 0.46
p = 0.32
Relative LAMP-2 intensity
E
Vehicle
CQ
NC
Counts
FL1-H (LC3B)
Relative autophagy flux (LC3 B MFI difference)
F
p-Tau (Ser404)
Tau-1
Merged
Manders overlap coefficient of Tau-1+ and p-Tau+

Supplementary Figure 2. Glial cell differentiation, lysosomal marker expression, and co-localization of Tau proteins in controls and MPS II-iPSC derived neurons. Related to Figure 1.

A IF images represent GFAP-positive cells from 4-week differentiated HC-, ISO-, and MPS II-iPSCs. DAPI was used to stain nuclei (blue); Scale bar: 50 μm. Histogram reveals differences in GFAP-positive cell percentages. N = 3 independent differentiations from 3 HC clones, N = 4 independent differentiations from 4 ISO clones from 2 patients, and N = 9 differentiation from 8 MPS II patients' clones. **B**, **C** Comparison of IDS activity (**B**) and GAG accumulation (**C**) across 0, 4, and 15 weeks in HC, ISO, and MPS II neurons. Left: IDS activity assay; Right: DMMB assay for GAG content. N = 3 independent differentiations from 3 HC clones, N = 4 independent differentiations from 4 ISO clones from 2 patients, and N = 8 differentiation from 8 MPS II patients' clones. **D** Western blotting analysis of LAMP-1 and LAMP-2 expression in 15-week HC, ISO, and MPS II neurons. Data were normalized to that of HC. N = 6 independent differentiations from 3 HC clones, N = 6 independent differentiations from 4 ISO clones from 2 patients, and N = 24 differentiation from 10 MPS II patients' clones. **E** After 24 hours of chloroquine treatment, 15-week MPS II neurons showed a significant reduction in autophagy flux compared to control neurons. Representative flow cytometry plots are shown (left panels). Autophagy flux was determined by calculating the difference in mean fluorescence intensities (MFI) of LC3B with chloroquine and vehicle treatment, with each subtracted from their respective negative control (NC: 2nd antibody only) MFIs. Data were normalized to that of HC and shown in a histogram (right panel). N = 4 independent differentiations from 3 HC clones, N = 4 independent differentiations from 3 ISO clones from 2 patients, and N = 8 differentiations from 8 MPS II patients' clones. **F** Double IF microscopy indicated co-localization of Tau-1 and p-tau in 15-week neurons. The histogram shows the variance in co-localization, as measured by Manders' overlap coefficient. N = 3

independent differentiations from 3 HC clones, N = 3 independent differentiations from 3 ISO clones from 2 patients, and N = 9 differentiations from 8 MPS II patients' clones. DAPI was used to stain nuclei (blue); Scale bar: 20 μm. All data are presented as mean ± SEM. Statistical significance was determined using the Mann-Whitney U test, between HC or ISO neurons and MPS II neurons unless indicated otherwise. ns: not significant; *$p < 0.05$, **$p < 0.01$, ***$p < 0.005$. Each data point presented originates from independent differentiations. Cell lines and clone details are in Supplementary Table 1.

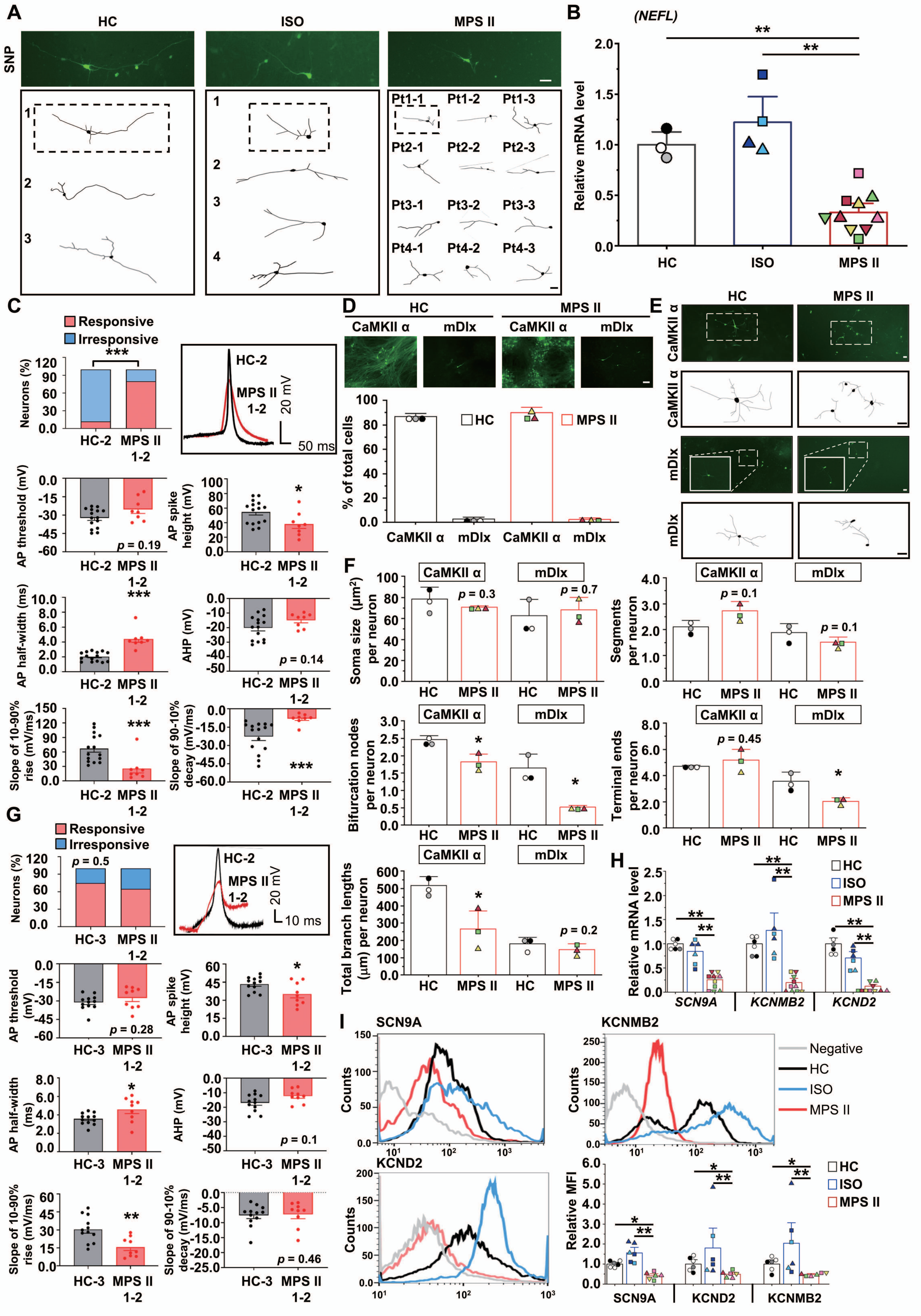
A
HC
ISO
MPS II
SNP
Pt1-1
Pt1-2
Pt1-3
Pt2-1
Pt2-2
Pt2-3
Pt3-1
Pt3-2
Pt3-3
Pt4-1
Pt4-2
Pt4-3
B
(NEFL)
Relative mRNA level
C
Responsive
Irresponsive
Neurons (%)
HC-2
MPS II 1-2
AP threshold (mV)
p = 0.19
AP spike height (mV)
AP half-width (ms)
AHP (mV)
p = 0.14
Slope of 10-90% rise (mV/ms)
Slope of 90-10% decay (mV/ms)
20 mV
50 ms
D
CaMKII α
mDlx
% of total cells
E
F
Soma size (μm²) per neuron
p = 0.3
p = 0.7
Segments per neuron
p = 0.1
Bifurcation nodes per neuron
Terminal ends per neuron
p = 0.45
Total branch lengths (μm) per neuron
p = 0.2
G
p = 0.5
HC-3
10 ms
p = 0.28
p = 0.1
p = 0.46
H
SCN9A
KCNMB2
KCND2
I
Counts
Negative
Relative MFI

Supplementary Figure 3. Neuronal phenotypes of MPS II-iPSC-derived excitatory (CaMKII α) and inhibitory (mDlx) neurons and distinct expression profiles of ion channel and neurodegeneration markers. Related to Figures 2 and 3.

A Neurite morphology of 15-week HC, ISO, and MPS II neurons visualized via fluorescent microscopy. In the lower panels, dashed rectangles highlight representative neurons transduced with hSYN-EGFP-AAV. Images were reconstructed using Neurolucida. Scale bar, 20 μm. N = 3 HC clones, N = 4 ISO clones from 2 patients, and N = 12 clones from 4 MPS II patients. Scale bar: 20 μm. **B** qRT-PCR demonstrated decreased *NEFL* mRNA levels in 15-week MPS II neurons. N = 3 HC clones, N = 4 from 4 ISO clones from 2 patients, and N = 10 MPS II clones from 4 patients. **C** Abnormal action potentials (AP) in 18-week mature MPS II neurons compared to HC neurons. These data correspond to the second set of data presented in Fig. 2C. We analyzed AP responses to current steps ranging from -50 to 150 pA. First, a decreased response rate to current injections in MPS II compared to HC was observed (left upper). N = 17 cells from 2 independent differentiations from HC-2 clones, N = 41 cells from 4 independent differentiations from MPS II 1-2 clone. $***p < 0.005$ (chi-square test). Second, representative traces depicted AP variations in MPS II and controls, with 20 mV and 50 ms scale bars. Histograms show the differences of AP parameters between MPS II and HC neurons, including AP threshold, AP spike height, AP half-width, after-hyperpolarization (AHP), slope of 10-90% rise, and slope of 90-10% decay. N = 15 cells from 2 independent differentiations from HC-2 clone, N = 8 cells from 4 independent differentiations from MPS II 1-2 clone. **D** At 20 weeks, MPS II and HC neurons displayed similar compositions of neuron subtypes. Representative IF images (upper panel) displayed the HC and MPS II neurons transduced with excitatory (*CaMKII α-EYFP*) and inhibitory (*mDlx-GFP*) neuron-specific AAVs. The histogram (lower panel) indicates that most of both groups were excitatory

neurons, with no significant difference in proportions. N = 3 independent differentiations from 3 HC clones, and N = 3 differentiation from 3 MPS II patients' clones. Scale bar, 50 μm. **E** Neurite morphology in 20-week MPS II neurons showed alterations compared to HC. Both were transduced with either *CaMKII α-EYFP* or *mDlx-GFP* AAVs. Neuron morphology is shown via fluorescence (upper panel) and reconstructed using Neurolucida (lower panel). Dashed rectangles highlight key neurons; insets magnify selected areas. **F** Neurite morphometrics in 20-week MPS II excitatory (CaMKII α) and inhibitory (mDlx) neurons. Parameters: soma size, segments, bifurcation nodes, terminal end, and branch length. N = 3 independent differentiations from 3 HC clones, and N = 3 differentiations from 3 MPS II patients' clones. *$p < 0.05$, analyzed by Student's t-test. Scale bar, 20 μm. **G** Abnormal action potentials (AP) in 22-week MPS II excitatory neurons compared to HC excitatory neurons. We analyzed AP responses to current steps ranging from -50 to 150 pA. First, a decreased response rate to current injections in MPS II compared to HC was observed (left upper). N = 16 cells from 3 independent differentiations from HC-3 clone, N = 14 cells from 2 independent differentiations from MPS II 1-2 clone. $p = 0.5$ (chi-square test). Second, representative traces depicted AP variations in MPS II and controls, with 20 mV and 10 ms scale bars. Histograms show the differences of AP parameters between MPS II and HC neurons, including AP threshold, AP spike height, AP half-width, AHP, slope of 10-90% rise, and slope of 90-10% decay. N = 12 cells from 3 independent differentiations from HC-3 clone, N = 9 cells from 2 independent differentiations from MPS II 1-2 clone. **H, I** qRT-PCR (**H**) and flow cytometry (**I**) analyses showed reduced levels of SCN9A, KCNMB2, and KCND2 mRNA and proteins in 15-week MPS II neurons. N = 6 from 3 HC clones, N = 6 from 4 ISO clones from 2 patients, and N = 10 (qRT-PCR); 6 (flow cytometry) MPS II clones from 4 patients.Statistics: Unless specified, data represent mean ± SEM. *$p < 0.05$, **$p < 0.01$, ***$p < 0.005$. Mann-Whitney U test. Each

data point presented originates from independent differentiations. Cell lines and clone details are in Supplementary Table 1.

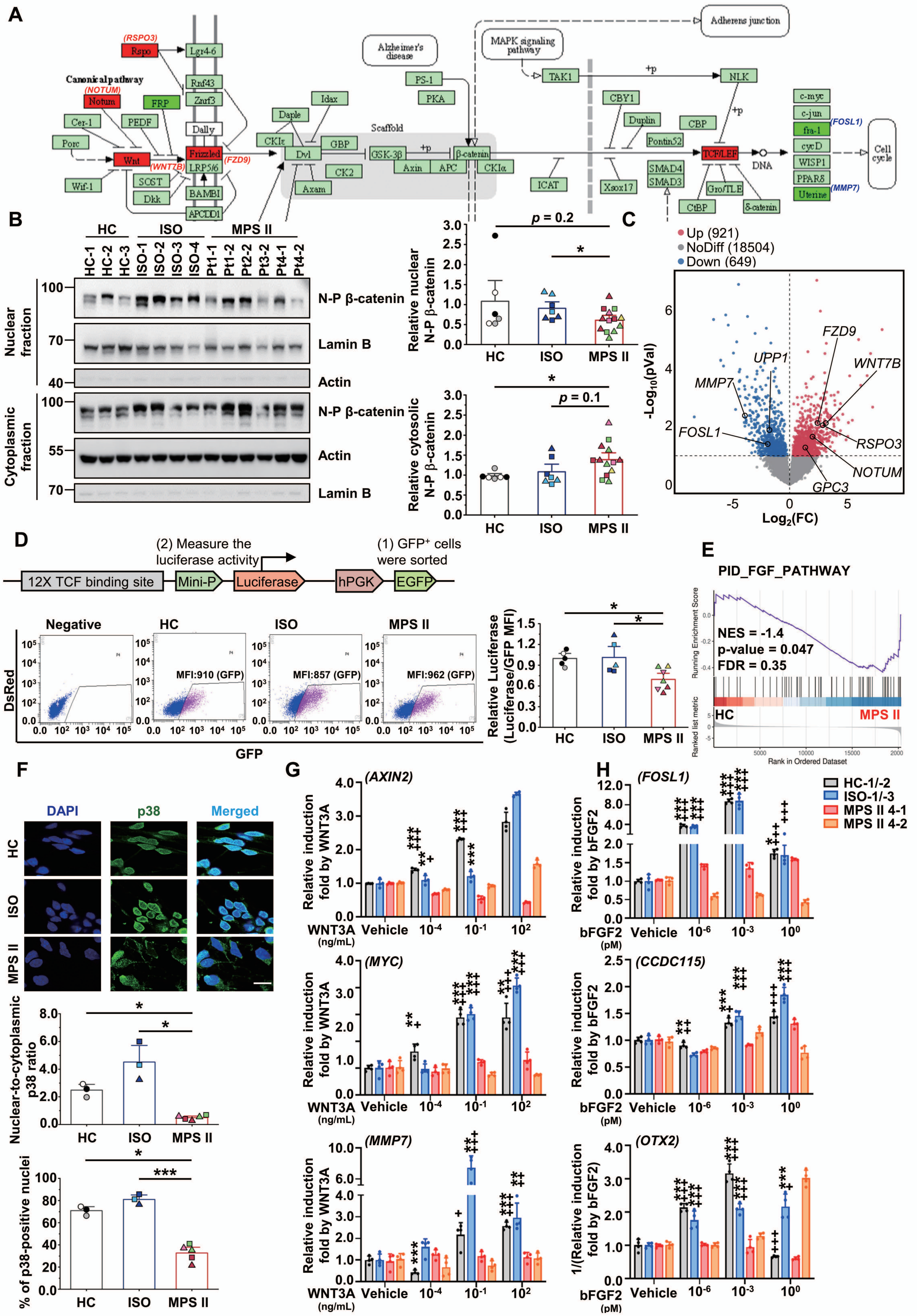
A
Canonical pathway
Rspo
Notum
Wnt
Frizzled
LRP5/6
Dvl
GSK-3β
Axin
APC
β-catenin
TCF/LEF
DNA
Cell cycle
Adherens junction
MAPK signaling pathway
Alzheimer's disease
Scaffold
B
HC
ISO
MPS II
N-P β-catenin
Lamin B
Actin
Nuclear fraction
Cytoplasmic fraction
Relative nuclear N-P β-catenin
Relative cytosolic N-P β-catenin
p = 0.2
p = 0.1
C
Up (921)
NoDiff (18504)
Down (649)
-Log10(pVal)
Log2(FC)
FZD9
UPP1
WNT7B
MMP7
FOSL1
RSPO3
NOTUM
GPC3
D
(2) Measure the luciferase activity
(1) GFP+ cells were sorted
12X TCF binding site
Mini-P
Luciferase
hPGK
EGFP
Negative
MFI:910 (GFP)
MFI:857 (GFP)
MFI:962 (GFP)
DsRed
GFP
Relative Luciferase (Luciferase/GFP MFI)
E
PID_FGF_PATHWAY
NES = -1.4
p-value = 0.047
FDR = 0.35
Running Enrichment Score
Ranked list metric
Rank in Ordered Dataset
F
DAPI
p38
Merged
Nuclear-to-cytoplasmic p38 ratio
% of p38-positive nuclei
G
(AXIN2)
(MYC)
(MMP7)
Relative induction fold by WNT3A
WNT3A (ng/mL)
Vehicle
H
(FOSL1)
(CCDC115)
(OTX2)
Relative induction fold by bFGF2
1/(Relative induction fold by bFGF2)
bFGF2 (pM)
HC-1/-2
ISO-1/-3
MPS II 4-1
MPS II 4-2

Supplementary Figure 4. Dysregulation of Wnt and p38 MAPK signaling in MPS II neurons. Related to Figures 3 and 4.

A In the KEGG Wnt signaling pathway (hsa04310), genes differentially regulated between 15-week MPS II and HC neurons are illustrated. Genes colored in red are upregulated, and those in dark green are downregulated. Most upregulated genes encode membrane or extracellular proteins, whereas downregulated genes largely represent TCF/LEF downstream targets. **B** An altered distribution of non-phosphorylated (active-form) β-catenin, characterized by decreased nuclear and increased cytosolic levels, in 15-week MPS II neurons. Proteins from iPSC-derived neurons were analyzed by Western blotting (left panel), with signal intensities normalized to Lamin B (nuclear) and β-actin (cytoplasmic). N = 6 independent differentiations from 3 HC clones, N = 7 independent differentiations from 4 ISO clones from 2 patients, and N = 13 differentiations from 7 MPS II patients' clones. **C** A volcano plot highlights the DEGs between MPS II and HC neurons, focusing on Wnt-associated genes. (*NOTUM, WNT7B, FZD9, RSPO3, GPC3, UPP1, FOSL1,* and *MMP7*). **D** Wnt signaling activity was reduced in MPS II neurons compared to controls. A Wnt signaling reporter was created by cloning 12 copies of LEF1/TCF-binding elements [1] upstream to a luciferase cDNA in a lentiviral vector with a PGK-GFP marker. 15-week HC, ISO, and MPS II neurons were transduced with this reporter lentivirus. Neurons expressing the reporter were sorted via GFP fluorescence, and luciferase activity was measured. Luciferase values were normalized to GFP mean fluorescence intensity (MFI) and shown as fold change relative to HC neurons. N = 5 independent differentiations from 3 HC clones, N = 5 independent differentiations from 4 ISO clones from 2 patients, and N = 7 differentiation from 4 MPS II patients' clones. **E** GSEA analysis indicated a reduction in FGF signaling in MPS II neurons compared to controls. **F** IF illustrated p38-positive cells, the nuclear-to-cytoplasmic ratio of p38 MAPK

fluorescence intensity, and the percentage of p38-positive nuclei in 15-week neurons. Scale bar, 10 µm. N = 3 independent differentiations from 3 HC clones, N = 3 independent differentiations from 3 ISO clones from 2 patients, and N = 5 differentiations from 4 MPS II patients' clones. *$p < 0.05$, analyzed by Welch's test for fluorescence intensity data and chi-square test for nuclei-positive cell data. **G, H** Investigation of the dynamics of downstream gene activation in 15-week MPS II and HC neurons exposed to exogenous WNT3A (**G**) or bFGF2 (**H**). The 15-week neurons were treated with vehicle (DMSO) or different concentrations of WNT3A and bFGF2 for 24 h before RNA extraction and qRT-PCR. The mRNA levels were normalized to GAPDH and vehicle only and presented as the fold change relative to each corresponding mean of MPS II groups (MPS II clones 4-1 and 4-2). #The *OTX2* gene is known to be downregulated by bFGF2 signaling [2] and served as a positive control. To enable a more effective comparison with other upregulated genes, the fold change in *OTX2* gene expression was reversed and used as the y-axis. N = 4 independent differentiations from 2 HC clones, N = 4 independent differentiations from 2 ISO clones from 2 patients, and N = 4 differentiations from 2 MPS II clones from patient 4. *$p < 0.05$, **$p < 0.01$, ***$p < 0.005$, compared with MPS II 4-1; $^{+}p < 0.05$, $^{++}p < 0.01$, $^{+++}p < 0.005$, compared with MPS II 4-2; analyzed by Student's t-test. Unless specified otherwise, statistical analyses were performed using the Mann-Whitney U test. ns: not significant; *$p < 0.05$, **$p < 0.01$, ***$p < 0.005$. Each data point presented originates from independent differentiations. Cell lines and clone details are in Supplementary Table 1.

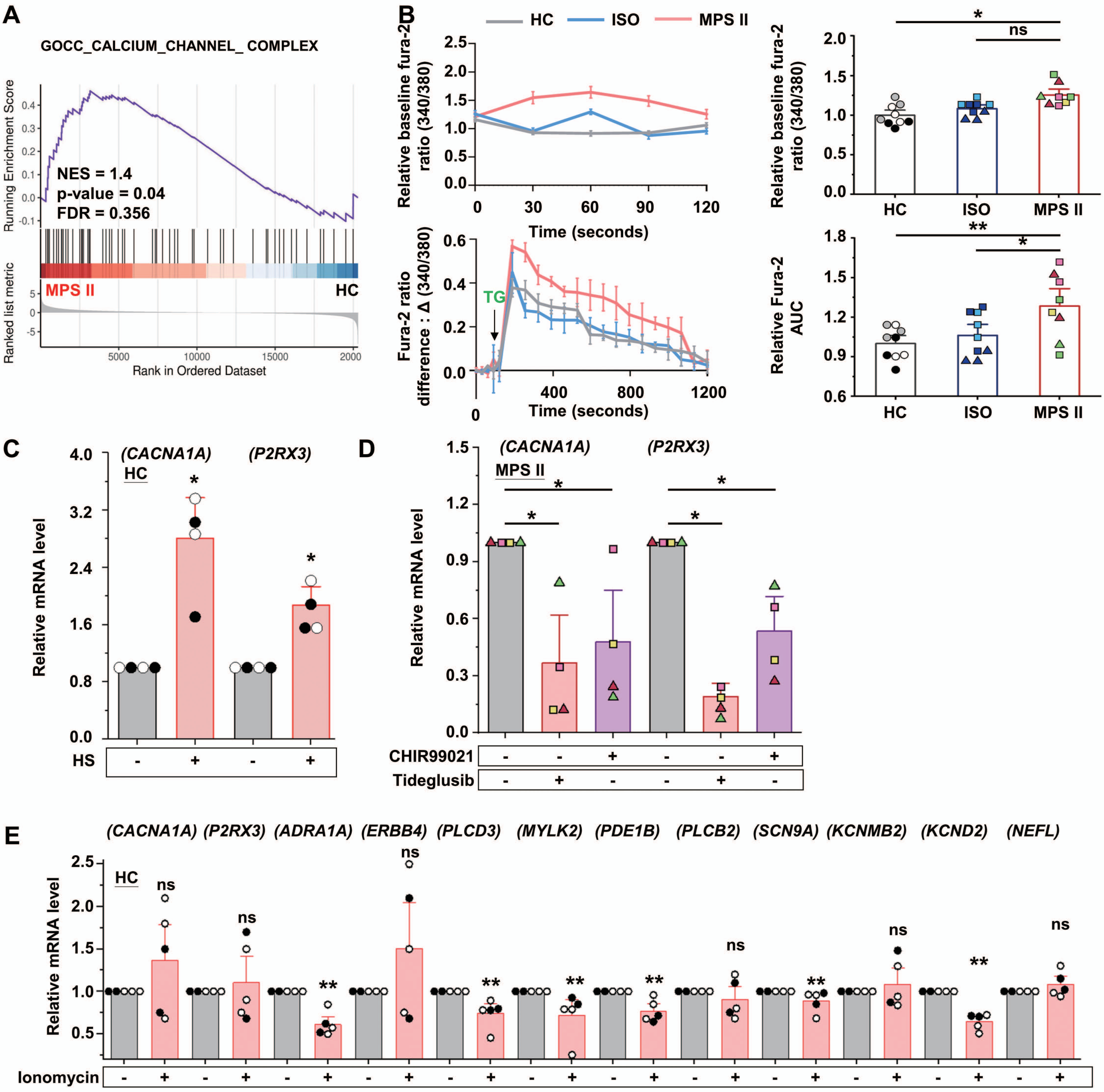
A
GOCC_CALCIUM_CHANNEL_ COMPLEX
Running Enrichment Score
NES = 1.4
p-value = 0.04
FDR = 0.356
Ranked list metric
MPS II
HC
Rank in Ordered Dataset
B
HC
ISO
MPS II
Relative baseline fura-2 ratio (340/380)
Time (seconds)
Fura-2 ratio difference : Δ (340/380)
TG
Relative Fura-2 AUC
ns
C
(CACNA1A)
(P2RX3)
Relative mRNA level
HS
D
CHIR99021
Tideglusib
E
(CACNA1A) (P2RX3) (ADRA1A) (ERBB4) (PLCD3) (MYLK2) (PDE1B) (PLCB2) (SCN9A) (KCNMB2) (KCND2) (NEFL)
Ionomycin

Supplementary Figure 5. Disrupted calcium homeostasis in MPS II neurons was revealed by spectrofluorometric analysis and the experimental approaches to elucidate the potential underlying molecular mechanisms. Related to Figure 4.

A GSEA revealed an enriched gene set associated with increased expression of calcium channel complex-encoding genes in MPS II neurons (see also Figure 4B). **B** Spectrofluorometric analysis to assess intracellular calcium changes in neurons following thapsigargin (TG) treatment. The alterations in the fluorescence intensity of the Fura-2 dye, were recorded at 340 nm and 380 nm wavelengths using a fluorescence microplate reader in 15-week neurons before and after TG treatment. Significantly higher baseline intracellular calcium levels, represented by the fluorescence intensity ratios at 340 nm and 380 nm (340/380), were observed in MPS II neurons compared to HC and ISO neurons (upper panels). Each dot in the upper histogram represents the mean ratio of 5 time points (0, 30, 60, 90, and 120 secs) for an individual clone or differentiation. The differences of fluorescence intensity ratios at 340 nm and 380 nm (340/380) between post-treatment and baseline, represented as "Δ (340/380)", were calculated. The area under the curve (AUC) represents the integral of Δ (340/380) fluorescence ratios plotted against time over a 20-minute duration, providing a quantitative measure of changes in intracellular calcium concentration (lower panels). Arrow: the time when TG was added. N = 9 independent differentiations from 3 HC clones, N = 9 independent differentiations from 3 ISO clones from 2 patients, and N = 8 differentiation from 5 MPS II clones from 4 patients. Data are presented as mean ± SEM. **C** Treatment of excessive heparan sulfate (HS) in 15-week HC neurons caused the upregulation of *CACNA1A* and *P2RX3*. N = 4 independent differentiations from 2 HC clones. **D** Treatment with GSK3 beta inhibitors Tideglusib and CHIR99021 resulted in reduced expression of calcium channel-regulating genes *CACNA1A* and *P2RX3* in 15-week MPS

II neurons, as shown in Figure 4A. The 15-week MPS II neurons were treated with Tideglusib or CHIR99021 for 24 hrs. N = 4 independent differentiations from 4 MPS II patients' clones. **E** Ionomycin treatment in 15-week HC neurons elevated intracellular calcium levels, inducing gene expression changes similar to those in MPS II neurons, with a significant reduction in the expression of calcium signaling-related genes (*ADRA1A*, *PLCD3, MYLK2,* and *PDE1B*) and other channel-encoding genes (*SCN9A* and *KCND2*). Significant effects were not observed for other calcium signaling-related genes, *CACNA1A*, *P2RX3*, *ERBB4*, and *PLCB2*. N = 5 independent differentiations from 2 HC clones. All data are presented as mean plus SEM. ns: not significant; *$p < 0.05$, **$p < 0.01$, ***$p < 0.005$. Cell lines and clone details are in Supplementary Table 1.

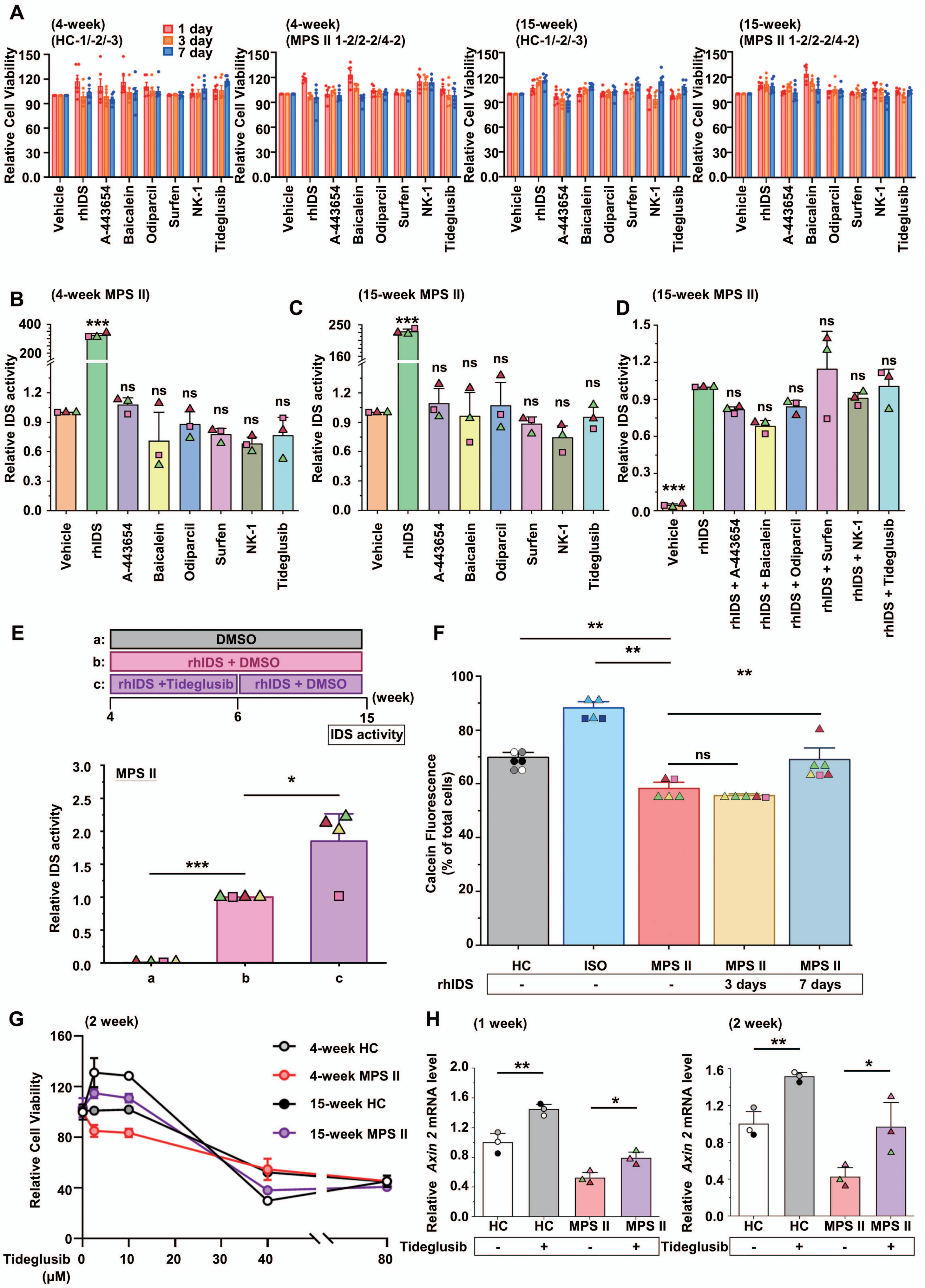
A
(4-week)
(HC-1/-2/-3)
1 day
3 day
7 day
Relative Cell Viability
Vehicle
rhIDS
A-443654
Baicalein
Odiparcil
Surfen
NK-1
Tideglusib
(4-week)
(MPS II 1-2/2-2/4-2)
(15-week)
(HC-1/-2/-3)
(15-week)
(MPS II 1-2/2-2/4-2)
B
(4-week MPS II)
C
(15-week MPS II)
D
(15-week MPS II)
Relative IDS activity
rhIDS + A-443654
rhIDS + Baicalein
rhIDS + Odiparcil
rhIDS + Surfen
rhIDS + NK-1
rhIDS + Tideglusib
E
a: DMSO
b: rhIDS + DMSO
c: rhIDS +Tideglusib | rhIDS + DMSO
(week)
4 6 15
IDS activity
MPS II
F
Calcein Fluorescence (% of total cells)
HC
ISO
MPS II
rhIDS
3 days
7 days
G
(2 week)
4-week HC
4-week MPS II
15-week HC
15-week MPS II
Tideglusib (μM)
H
(1 week)
(2 week)
Relative Axin 2 mRNA level
Tideglusib

Supplementary Figure 6. Impact of selected drug dosages on neuron survival and IDS activity in iPSC-derived neurons. Related to Figures 5 and 6.

A Evaluation of cytotoxicity in 4-week and 15-week HC and MPS II neurons treated with various drugs. Drugs and dosages: rhIDS (20 ng/ml), A-443654 (0.05 μM), baicalein (10 μM), Odiparcil (10 μM), surfen (10 μM), NK-1 (0.5 μM), and Tideglusib (10 μM). Cell viability was measured using the XTT assay after 1-, 3-, and 7-day drug exposure. The value of each drug was normalized to that of the corresponding vehicle. N = 6 independent differentiations from 3 HC clones, and N = 6 differentiation from 3 MPS II patients' clones. **B-D** The changes of IDS activity in 4-week (**B**) and 15-week (**C**, **D**) MPS II neurons treated with individual drugs (**B**, **C**) or the rhIDS-drug combinations (**D**) for 7 days. In (**B**, **C**), the comparisons are made relative to the vehicle, whereas for (**D**), the comparisons were made relative to rhIDS. N = 3 from 3 MPS II patients' clones. **E** Neurons initially treated with a combined regimen of rhIDS and Tideglusib from week 4 to week 6, followed by ongoing rhIDS treatment until week 15, exhibited significantly enhanced IDS activity compared to neurons treated with rhIDS alone. N = 4 differentiation from 4 MPS II patients' clones. These groups were compared to MPS II neurons treated with rhIDS only. **F** The effect of 3 days or 7 days of rhIDS treatment on the viability of MPS II neurons was evaluated using calcein-AM flow cytometry. Combinatory effects of rhIDS and different drugs in 4-week neurons are shown in Fig. 5B. N = 6 independent differentiations from 3 HC clones, N = 5 independent differentiations from 2 ISO clones from 2 patients, and N = 5-6 differentiations from 4 MPS II patients' clones. These groups were compared to MPS II neurons treated with PBS. **G** Analysis of Tideglusib cytotoxicity following a 2-week treatment period in 4-week and 15-week HC and MPS II neurons, with respective CC50 values provided. N = 6 independent differentiations from 3 HC clones, and N = 6 differentiations from 3 MPS II patients' clones. **H** Tideglusib (10 μM)

treatment for 1 (left panel) and 2 (right panel) weeks induced significant expression of *AXIN2*, a Wnt signaling target [3], in both MPS II and HC neurons, assayed by qRT-PCR. N = 3 differentiations from 3 MPS II patients' clones and N = 3 differentiations from 3 HC clones. All data are presented as mean ± SEM. ns: not significant; $*p < 0.05$, $**p < 0.01$, $***p < 0.005$. Student's t-test was used for (**B**-**D**) and (**H**), while the Mann-Whitney U test was applied for all other data. Each data point presented originates from independent differentiations. Cell lines and clone details are in Supplementary Table 1.

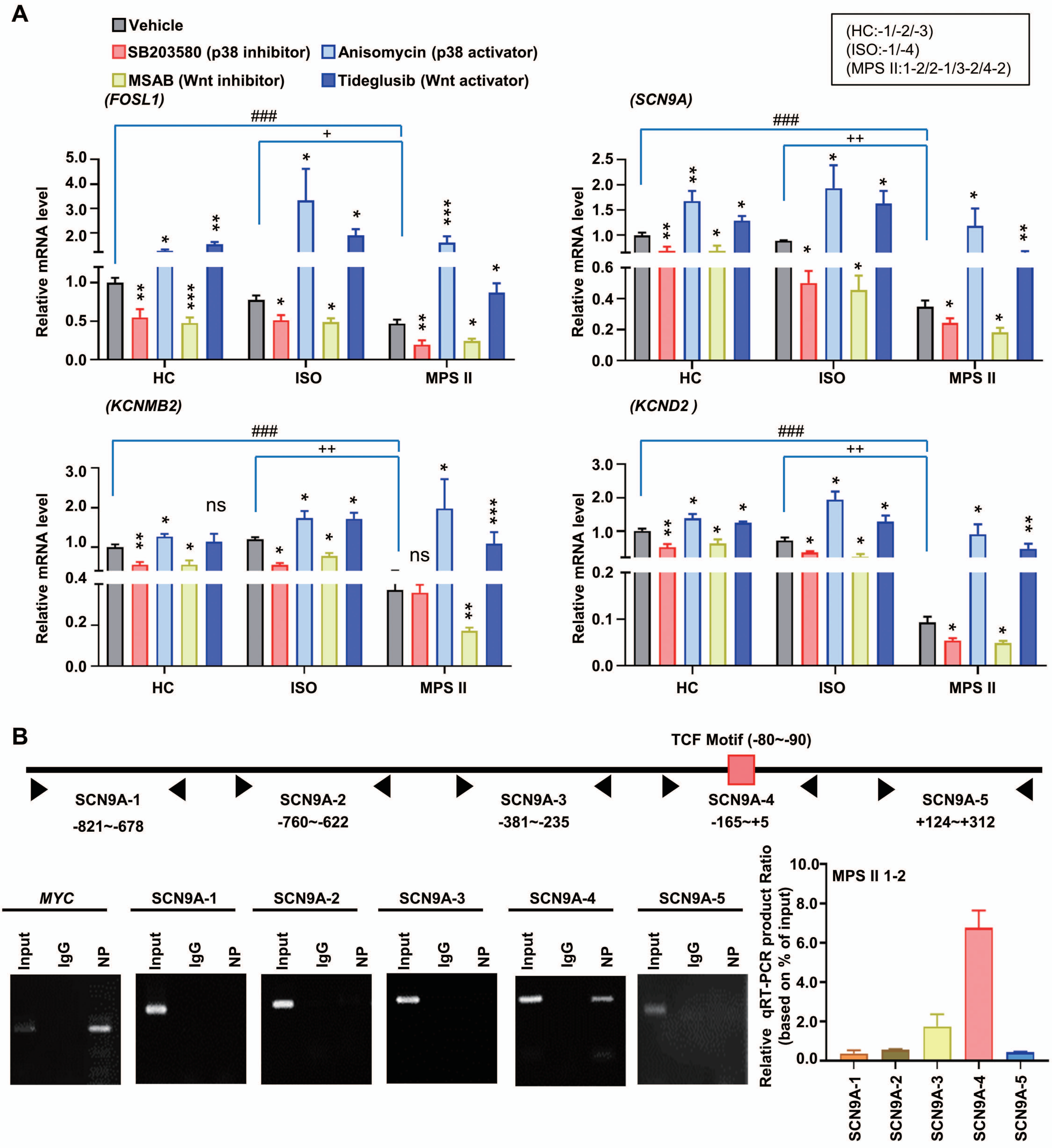
A
Vehicle
SB203580 (p38 inhibitor)
Anisomycin (p38 activator)
MSAB (Wnt inhibitor)
Tideglusib (Wnt activator)
(HC:-1/-2/-3)
(ISO:-1/-4)
(MPS II:1-2/2-1/3-2/4-2)
(FOSL1)
(SCN9A)
(KCNMB2)
(KCND2)
Relative mRNA level
HC
ISO
MPS II
ns
B
TCF Motif (-80~-90)
SCN9A-1
-821~-678
SCN9A-2
-760~-622
SCN9A-3
-381~-235
SCN9A-4
-165~+5
SCN9A-5
+124~+312
MYC
Input
IgG
NP
MPS II 1-2
Relative qRT-PCR product Ratio (based on % of input)

Supplementary Figure 7. Modulation of Wnt/β-catenin, p38 signaling, and ion channel genes by different small molecules in control and MPS II neurons; β-catenin's binding to SCN9A promoter. Related to Figure 7.

A Expression levels of *FOSL1* and ion channel-encoding genes (*SCN9A*, *KCNMB2*, *KCND2*) were modulated by p38 and Wnt/β-catenin signaling activators and inhibitors. The 15-week HC, ISO, and MPS II neurons were treated with vehicle (DMSO), 25 μM SB203580 (p38 inhibitor) [4], 0.1 μM anisomycin (p38 activator) [5], 5 μM MSAB (Wnt/β-catenin signaling inhibitor) [6], or 10 μM Tideglusib (Wnt/β-catenin signaling activator) for 24 h before RNA extraction and qRT-PCR. The mRNA levels were normalized to GAPDH and presented as the fold change relative to HC. *FOSL1* is a well-known downstream target gene of both Wnt/β-catenin [7] and p38 [8] signaling pathways. Note that the mRNA levels of these four genes were significantly lower in MPS II than in HC and ISO neurons. N = 7 independent differentiations from 3 HC clones, N = 4 independent differentiations from 2 ISO clones of 2 MPS II patients, and N = 7 differentiations from 4 MPS II patients' clones. ns: not significant; $^{*}p < 0.05$, $^{**}p < 0.01$, $^{***}p < 0.005$, compared to vehicle control; $^{\#}p < 0.05$, $^{\#\#}p < 0.01$, $^{\#\#\#}p < 0.005$, compared to HC; $^{++}p < 0.01$, $^{+++}p < 0.005$, compared to ISO; analyzed by Mann-Whitney U test. **B** Chromatin immunoprecipitation (ChIP) assays demonstrated β-catenin binding to the LEF1/TCF motif within the *SCN9A* promoter region. ChIP assay was performed by both PCR (left, gel pictures) and qRT-PCR (right, histogram) using primers targeting the human *SCN9A* promoter regions. The upper line indicates the promoter of *SCN9A*. The position +1 indicates the transcription start site. The red rectangle represents the presumed location of the LEF1/TCF binding motif found by JASPAR online software (https://jaspar.genereg.net/analysis), with black triangles marking other randomly selected regions (SCN9A1~5) on both sides, which were the targets of PCR. A ChIP assay

was performed as described in detail in Methods. Briefly, sonicated DNA fragments from 15-week neurons were immunoprecipitated by anti-non-phosphorylated β-catenin antibody or IgG (negative control) and then subjected to PCR and qRT-PCR to analyze the relative enrichment of DNA fragments around the putative LEF1/TCF site. A well-known LEF1/TCF site in the *MYC* promoter [9] was used as a positive control (the first gel). PCR and qRT-PCR were performed in biological triplicates, and similar results were obtained. The images of agarose gel electrophoresis show PCR results, while the histogram shows qRT-PCR results. Cell lines and clone details are in Supplementary Table 1.

Supplementary Table 1. Detailed information of clones used in various figures.

Figure legend number	Names of clone	Numbers of clones for MPS II	Notes
**Figure 1A-E**	HC-1, -2, -3; ISO-1, -2, -3, 4; MPS II 1-1, 1-2,1-3, 2-1, 2-2, 2-3, 3-1 ,3-2, 3-3,4-1,4-2, 4-3	12 clones from 4 patients	
**Figure 1F. G**	HC-1, -2, -3; ISO-1, -2, -3, 4; MPS II 1-1, 1-2,1-3, 2-1, 2-2, 3-1 ,3-2, 4-1, 4-3	9 clones from 4 patients	n= 200 cells from 5 fields for each clone
**Figure 2A**	HC-1, -2, -3; ISO-1, -2, -3, 4; MPS II 1-1, 1-2,1-3, 2-1, 2-2, 2-3, 3-1 ,3-2, 3-3,4-1,4-2, 4-3	12 clones from 4 patients	n=50 cells for each clone
**Figure 2B**	HC-1, -2, -3; ISO-1, -2, -3, 4; MPS II 1-2,2-1,2-2,3-3,4-1,4-2	6 clones from 4 patients	
**Figure 2C**	HC-1, ISO-4/ MPS II 4-1	3 independent differentiations	n= 14 (12) cells and n=15 (13) cells from 4 independent differentiations for HC-1 and ISO-4 each; n=24 (6) cells from 3 independent differentiations for MPS II 4-1; numbers in parentheses: AP parameters data
**Figure 3A, B**	HC-1, -2, -3; MPS II 1-1, 1-2,1-3, 2-2, 2-3, 3-1, 3-3, 4-2, 4-3	9 clones from 4 patients	
**Figure 3C**	HC-1, -2; ISO-1, -2, -3, -4; MPS II 1-1,1-2,2-2,2-3, 4-1,4-2	6 clones from 3 patients	4 independent differentiations for HC-1 and HC-2 each; 2 independent differentiations for ISO-1~4 and MPS II each
**Figure 3D.**	HC-1, -2, -3; ISO-1, -2, -3, -4; MPS II 1-2,2-2,3-2,4-2	4 clones from 4 patients	2 independent differentiations for MPS II 1-2 and 2-2 each
**Figure 3E**	HC-1, -2, -3; ISO-1, -2, -3, -4; MPS II 1-2,2-2,3-2,4-2	4 clones from 4 patients	2 independent differentiations for ISO-1; MPS II 1-2, 2-2, and 4-2 each
**Figure 3F**	HC-1, -2, -3; ISO-1, -2, -3, -4; MPS II 1-2,2-3,4-2	3 clones from 3 patients	n= 20 cells from 5 fields for each clone
**Figure 4A, B**	HC-1, -2, -3; MPS II (w/o 2-1,3-2,4-1)	9 clones from 4 patients	
**Figure 4 C**	HC-1, -2, -3; ISO-1, -4; MPS II 1-2,2-2,3-2,4-2,4-3	5 clones from 4 patients	2 independent differentiations for HC-1,ISO-1, and ISO-4 each
**Figure 4 D. E**	HC-1, -2; ISO-1, -3; MPS II 1-2,2-2,4-2,4-3	4 clones from 3 patients	2 independent differentiations for each clone
**Figure 4 F**	MPS II 1-2, 2-1, 3-1,4-2	4 clones from 4 patients	
**Figure 5 A-F**	MPS II 1-2, 2-1,4-2	3 clones from 3 patients	
**Figure 5 G. H**	HC-2; ISO-4; MPS II 4-1,4-2	2 clones from patient 2	3 independent differentiations for each clone
**Figure 6A**	MPS II 1-2, 2-1,2-2,3-1, 4-1,4-2	6 clones from 4 patients	
**Figure 6B**	MPS II 1-1,1-2, 2-2,3-3,4-1,4-2	6 clones from 4 patients	2 independent differentiations for MPS II 1-2,4-1 and 4-2 each
**Figure 6C**	MPS II 1-2	More than 3 independent differentiations	n= 16 (12) cells from 3 independent differentiations for vehicle group; n= 14 (12) cells from 5 independent differentiations for Tideglusib group; numbers in parentheses: AP parameters data
**Figure 6D**	MPS II 1-1,1-2, 4-1,4-2	4 clones from 2 patients	n=50 cells for each clone
**Figure S1A-E**		12 clones from 4 patients	
**Figure S1F. G**	MPS II 1-2, MPS II 4-2	2 rescued clones from 2 patients	totally 4 isogenic controls
**Figure S2A**	HC-1, -2, -3; ISO-1, -2, -3, -4; MPS II 1-2,1-3, 2-1, 2-2, 3-2, 3-3, 4-1,4-2	8 clones from 4 patients	2 independent differentiations for MPS II 1-3
**Figure S2B.C**	HC-1, -2, -3; ISO-1, -2, -3, -4; MPS II 1-2,1-3, 2-1, 2-2, 3-2, 3-3, 4-1,4-2	8 clones from 4 patients	
**Figure S2D**	HC-1, -2, -3; ISO-1, -2, -3, 4; MPS II 1-1, 1-2,1-3, 2-1, 2-2, 3-1 ,3-2, 4-1,4-2, 4-3	10 clones from 4 patients	2 independent differentiations for HC-1,-2,-3; ISO-1,-2 and MPS II 1-2,1-3,3-1,4-3 each; 3 independent differentiations for MPS II 1-1, 2-1,2-2, 4-1 and 4-2 each
**Figure S2E**	HC-1, -2, -3; ISO-1, -2, -3; MPS II 1-2,1-3, 2-1, 2-2, 3-2, 3-3, 4-1,4-2	8 clones from 4 patients	2 independent differentiations for HC-1 and ISO-2 each
**Figure S2F**	HC-1, -2, -3; ISO-1, -3, -4; MPS II 1-2,1-3, 2-1, 2-2, 3-2, 3-3, 4-1,4-2	8 clones from 4 patients	2 independent differentiations for MPS II 1-3
**Figure S3A**	HC-1, -2, -3; ISO-1, -2, -3, 4; MPS II 1-1, 1-2,1-3, 2-1, 2-2, 2-3, 3-1 ,3-2, 3-3,4-1,4-2, 4-3	12 clones from 4 patients	
**Figure S3B**	HC-1, -2, -3; ISO-1, -2, -3, -4; MPS II 1-1,1-2,1-3, 2-1,2-2,3-2,3-3,4-1,4-2,4-3	10 clones from 4 patients	
**Figure S3C**	HC-2; MPS II 1-2	4 independent differentiations	n= 17 (15) cells from 2 independent differentiations for HC-2; n=41 (8) cells from 4 independent differentiations MPS II 1-2; numbers in parentheses: AP parameters data
**Figure S3D. E. F**	HC-1, -2, -3; MPS II 1-2, 3-2,4-1	3 clones from 3 patients	n=50 cells for each clone in panel F.
**Figure S3G**	HC-3; MPS II 1-2	2 independent differentiations	n=16 (12) cells from 3 independent differentiations for HC-3; n=14 (9) cells from 2 independent differentiations MPS II 1-2; numbers in parentheses: AP parameters data
**Figure S3H**	HC-1, -2, -3; ISO-1, -2, -3, -4; MPS II 1-2, 2-1,2-2, 3-3,4-1,4-2	6 clones from 4 patients	
**Figure S3I**	HC-1, -2, -3; ISO-1, -2, -3, -4; MPS II 1-1,1-2, 1-3, 2-1,2-2, 3-1,3-3,4-1,4-2, 4-3	10 clones from 4 patients	
**Figure S4A**	HC-1, -2, -3; MPS II 1-1, 1-2,1-3, 2-2, 2-3, 3-1, 3-3, 4-2, 4-3	9 clones from 4 patients	
**Figure S4B**	HC-1, -2, -3; ISO-1, -2, -3, -4; MPS II 1-1,1-2,2-1,2-2,3-2,4-1,4-2	7 clones from 4 patients	2 independent differentiations for HC-1,-2,-3, ISO-1,-2,-4, and MPS II 1-1,4-1 each; 3 independent differentiations for MPS II 1-2 and 4-2 each.
**Figure S4C**	HC-1, -2, -3; MPS II 1-1, 1-2,1-3, 2-2, 2-3, 3-1, 3-3, 4-2, 4-3	9 clones from 4 patients	
**Figure S4D**	HC-1, -2, -3; ISO-1, -2, -3, -4; MPS II 1-2,2-2,3-2,4-2	4 clones from 4 patients	2 independent differentiations for ISO-1 and MPS II 1-2, 2-3 4-2 each; 3 independent differentiations for HC-2.
**Figure S4F**	HC-1, -2, -3; ISO-1, -3, -4; MPS II 1-1,1-2,2-2,4-1,4-2	5 clones from 3 patients	n= 20 cells for each clone
**Figure S4 G.H**	HC-1, -2; ISO -1, -3; MPS II 4-1,4-2	2 clones from patient 2	4 independent differentiations for each clone
**Figure S5A**	HC-1, -2, -3; MPS II 1-1, 1-2,1-3, 2-2, 2-3, 3-1, 3-3, 4-2, 4-3	9 clones from 4 patients	
**Figure S5B**	HC-1, -2, -3; ISO-1, -2, -3; MPS II 1-2, 2-1, 3-1, 4-1,4-2	5 clones from 4 patients	2 independent differentiations for MPS II 1-2,2-1 and 4-1 each; 3 independent differentiations for HC-1,2,3 and ISO-1,-2, 3 each
**Figure S5C**	HC-1, -2		2 independent differentiations for each clone
**Figure S5D**	MPS II 1-2, 2-1, 3-1, 4-2	4 clones from 4 patients	
**Figure S5E**	HC-1, -2		2 independent differentiations for HC-2; 3 independent differentiations for HC-1
**Figure S6A**	HC-1, -2, -3; MPS II 1-2,2-2,4-2	3 clones from 3 patients	2 independent differentiations for each clone
**Figure S6B. C. D**	MPS II 1-2,2-1,4-2	3 clones from 3 patients	
**Figure S6E**	MPS II 1-2,2-1,3-2, 4-2	4 clones from 4 patients	
**Figure S6F**	HC-1, -2, -3; ISO-1, -4; MPS II 1-2,2-1,3-2, 4-2	4 clones from 4 patients	1 to 2 independent differentiations for MPS II 1-2; 2 independent differentiations for HC-1,-2,-3, ISO-1 and MPS II 4-2 each; 3 independent differentiations for ISO-4
**Figure S6G**	HC-1, -2, -3; MPS II 1-2,2-2,4-2	3 clones from 3 patients	2 independent differentiations for each clone
**Figure S6H**	HC-1, -2, -3; MPS II 1-2,2-2,4-2	3 clones from 3 patients	
**Figure S7A**	HC-1, -2, -3; ISO-1, -4; MPS II 1-2,2-1,3-2, 4-2	4 clones from 4 patients	2 independent differentiations for MPS II 1-2,2-1, and 4-2 each
**Figure S7B**	MPS II 1-2		

Supplementary Table 2. Significant changes in p38 MAPK and PI3K signaling in MPS II Neurons identified by IPA of DEGs

Upstream Regulator	Predicted Activation State	Activation z-score	p-value of overlap
p38 MAPK	Inhibited	-2.172	0.000216
PI3K (complex)	Inhibited	-2.003	0.0439

This table displays the results obtained from the Upstream Regulator Analysis within the Ingenuity Pathway Analysis (IPA).

Supplementary Table 3. Concentration of cytotoxicity 50% (CC50) values of selected drugs in 4-week and 15-week HC and MPS II neurons

(Day) / (μM)	1day	3day	7day
A-443654	> 1000/16/61/1.4	>1000/15.2/77/0.8	203/0.5/21/1.4
Baicalein	> 1000/121/> 1000/> 1000	> 1000/257/128/> 1000	167/25/91/> 1000
Odiparcil	> 1000/895/112/111	> 1000/322/106/112	364/308/105/107
Surfen	> 1000/> 1000/> 1000/162	103/47.9/454/105	64/86/62/70
NK-1	> 1000	> 1000	> 1000
Tideglusib	>1000/110/112/109	> 1000/212/106/96	245/663/112/103

(4-week HC CC_{50}/4-week MPSII CC_{50}/15-week HC CC_{50}/15-week MPS II CC_{50})

The CC50 values presented in this table were determined from iPSC-derived neurons treated with A-443654, Baicalein, Odiparcil, Surfen, NK-1, and Tideglusib for durations of 1, 3, or 7 days.

References

1. Green JL, Inoue T, Sternberg PW. Opposing Wnt pathways orient cell polarity during organogenesis. Cell. 2008;134(4):646-56.

2. Partanen J. FGF signalling pathways in development of the midbrain and anterior hindbrain. Journal of neurochemistry. 2007;101(5):1185-93.

3. Jho E-h, Zhang T, Domon C, Joo C-K, Freund J-N, Costantini F. Wnt/β-catenin/Tcf signaling induces the transcription of Axin2, a negative regulator of the signaling pathway. Molecular and cellular biology. 2002;22(4):1172-83.

4. Düzgün ŞA, Yerlikaya A, Zeren S, Bayhan Z, Okur E, Boyacı İ. Differential effects of p38 MAP kinase inhibitors SB203580 and SB202190 on growth and migration of human MDA-MB-231 cancer cell line. Cytotechnology. 2017;69:711-24.

5. Chen L, Zhou X, Kong X, Su Z, Wang X, Li S, et al. The prognostic significance of anisomycin-activated phospho-c-Jun NH2-terminal kinase (p-JNK) in predicting breast cancer patients' survival time. Frontiers in Cell and Developmental Biology. 2021;9:656693.

6. Hwang S-Y, Deng X, Byun S, Lee C, Lee S-J, Suh H, et al. Direct targeting of β-catenin by a small molecule stimulates proteasomal degradation and suppresses oncogenic Wnt/β-catenin signaling. Cell reports. 2016;16(1):28-36.

7. Zhu X-y, Du Q-x, Li S-q, Sun J-h. Comparison of the homogeneity of mRNAs encoding SFRP5, FZD4, and Fosl1 in post-injury intervals: subcellular localization of markers may influence wound age estimation. Journal of forensic and legal medicine. 2016;43:90-6.

8. Jiang X, Xie H, Dou Y, Yuan J, Zeng D, Xiao S. Expression and function of FRA1 protein in tumors.

Molecular Biology Reports. 2020;47(1):737-52.

9. He T-C, Sparks AB, Rago C, Hermeking H, Zawel L, Da Costa LT, et al. Identification of c-MYC as a target of the APC pathway. Science. 1998;281(5382):1509-12.
