# Supplementary material for: Mature neurons from iPSCs unveil neurodegeneration-related pathways in mucopolysaccharidosis type II: GSK-3β inhibition for therapeutic potential
Source: Cell Death Dis. 2024 Apr 29;15(4):302. doi: 10.1038/s41419-024-06692-9 (PMC11058230; doi:10.1038/s41419-024-06692-9)

Supplementary information

original full-length Western blots

original full-length Western blots presented in Fig. 3C

p-GSK-3β

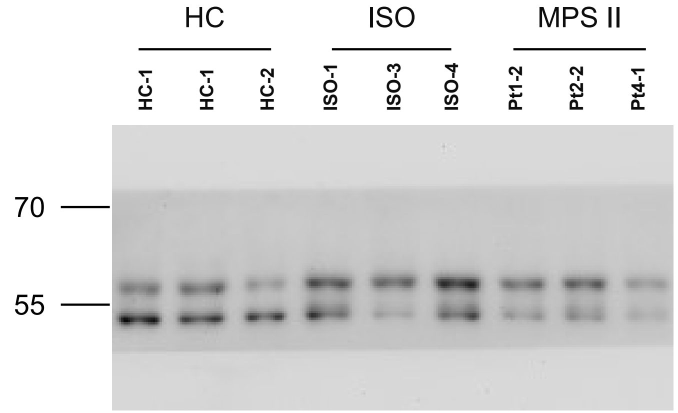

p-β-catenin

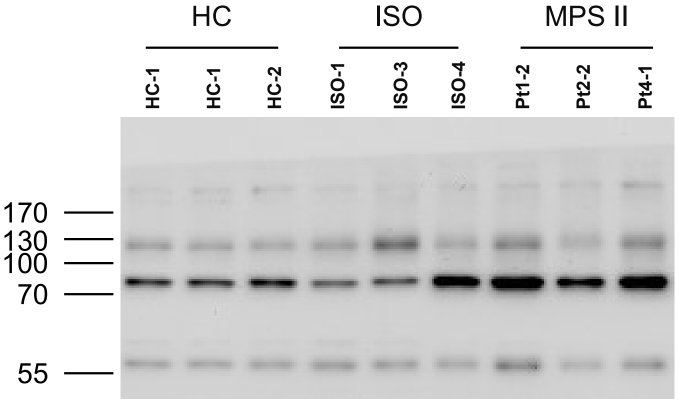

GAPDH

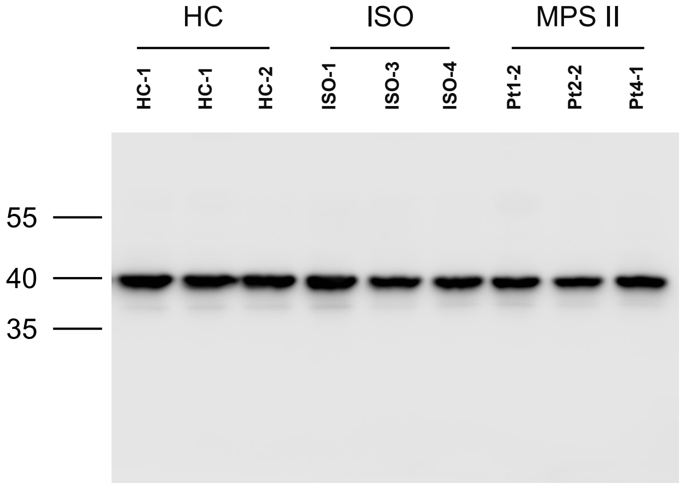

Total GSK-3β

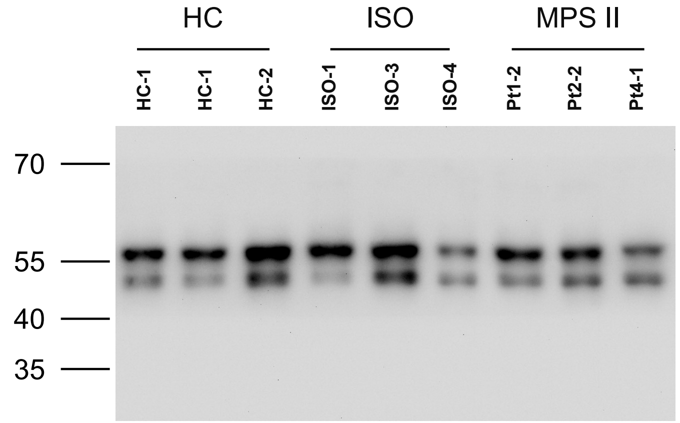

Total β-catenin

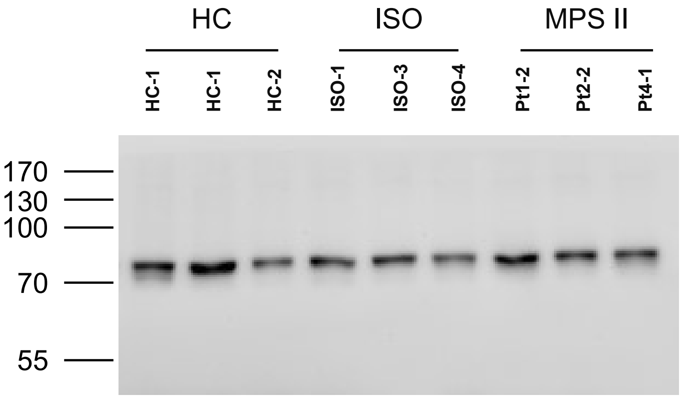

Additional blots for quantifications in Fig. 3C: The green box indicates the protein of interest.

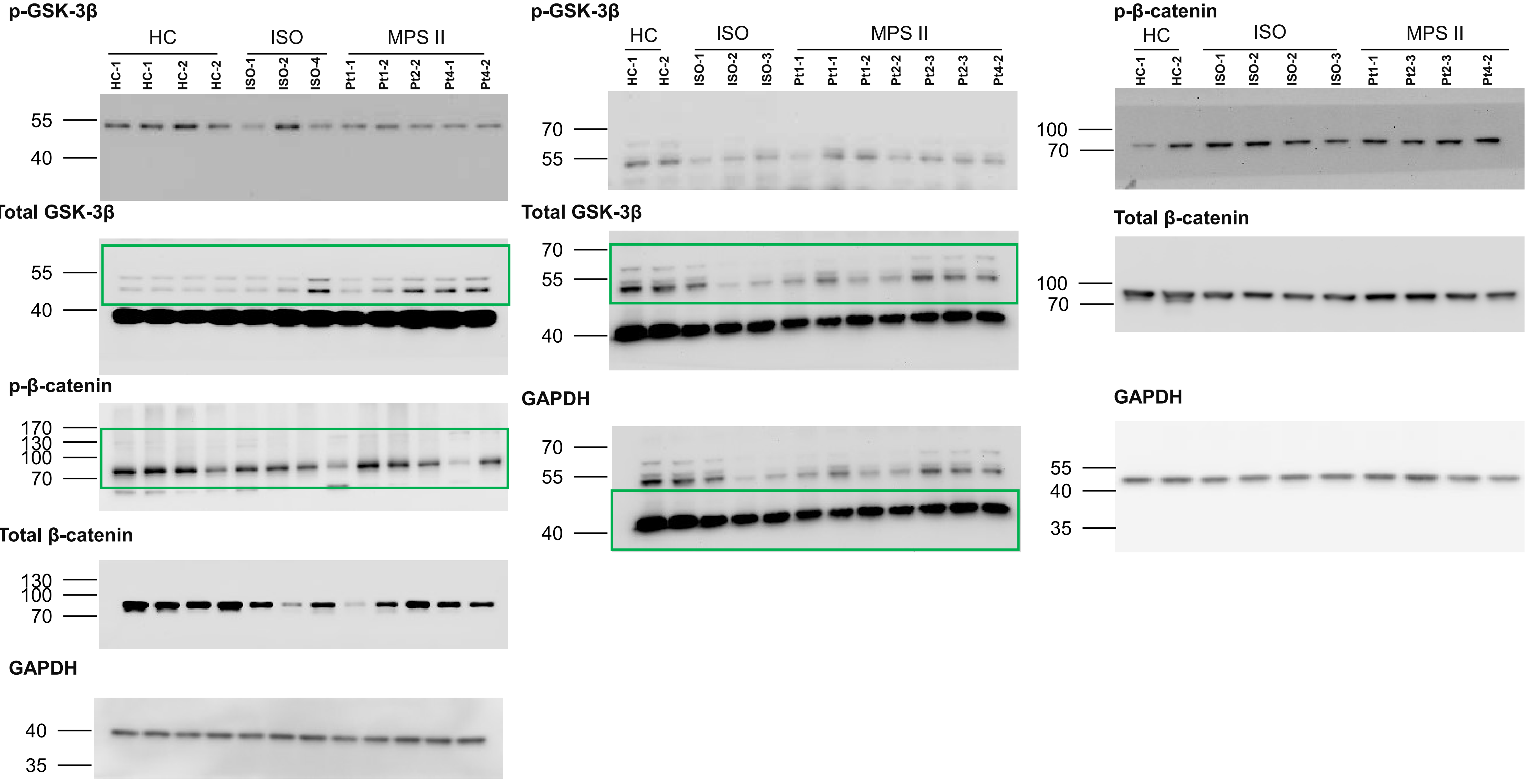

Supplementary figure

original full-length Western blots presented in Supplementary Fig. 2D

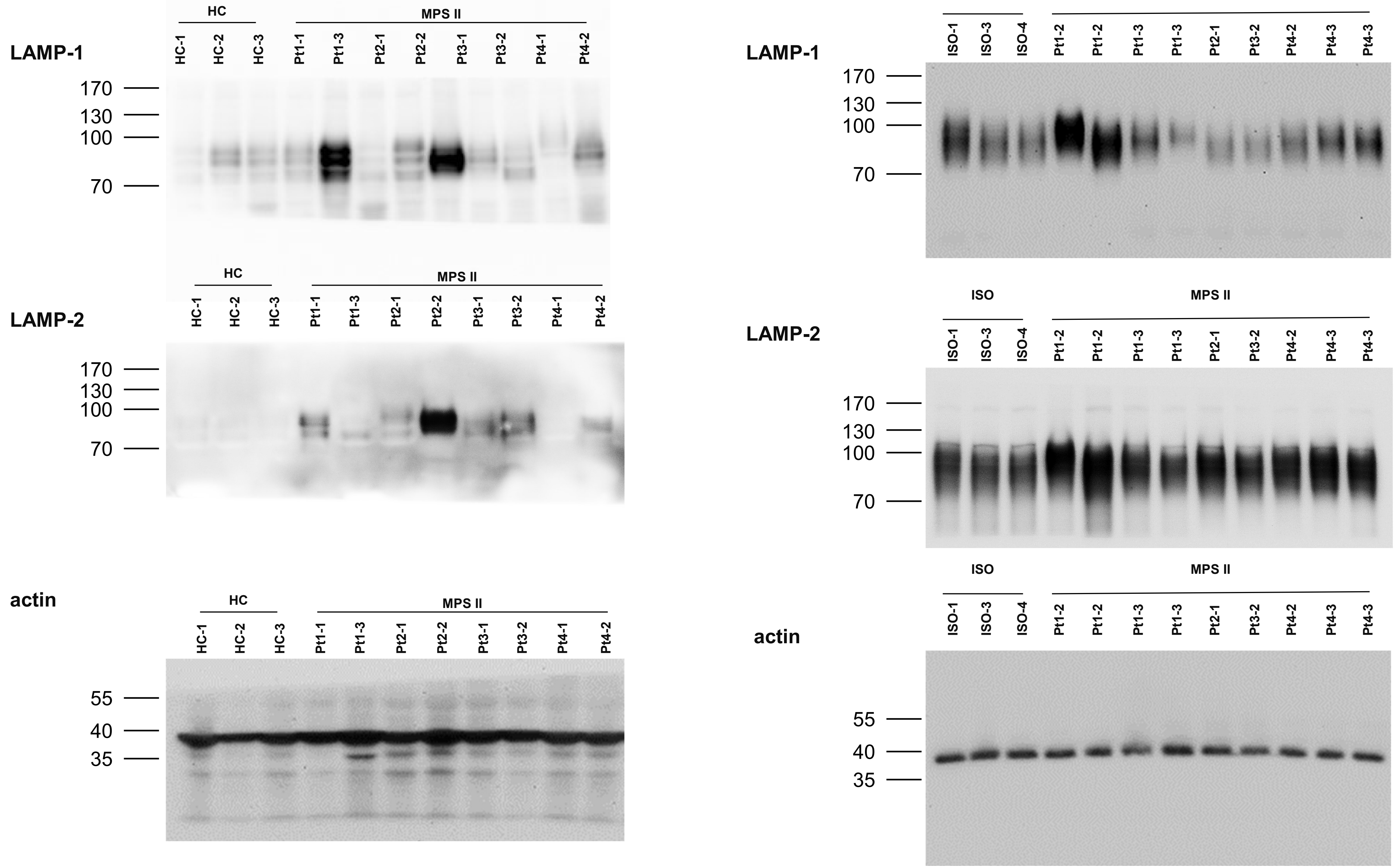

Additional blots for quantifications in Supplementary Fig. 2D.

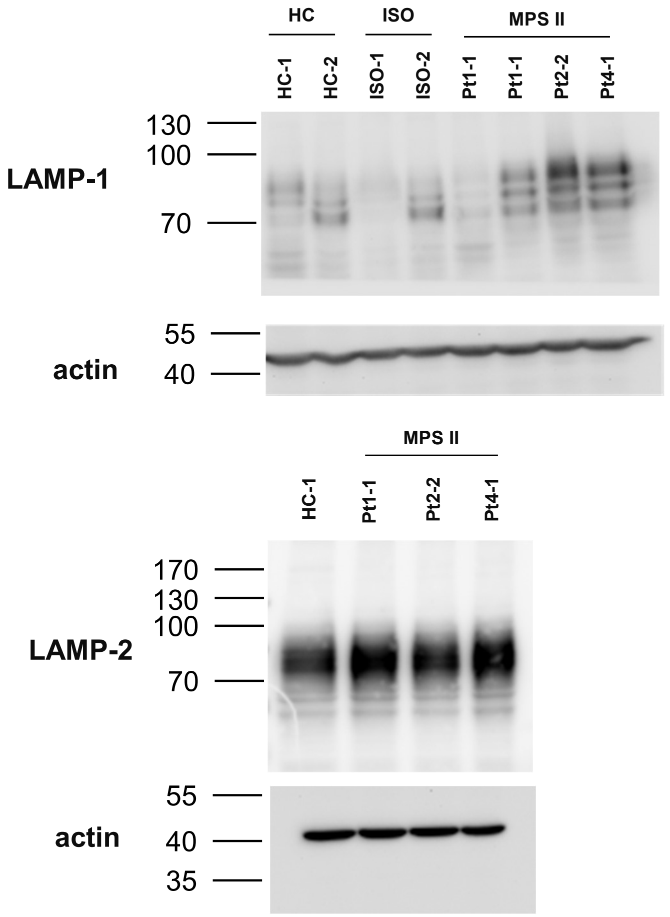

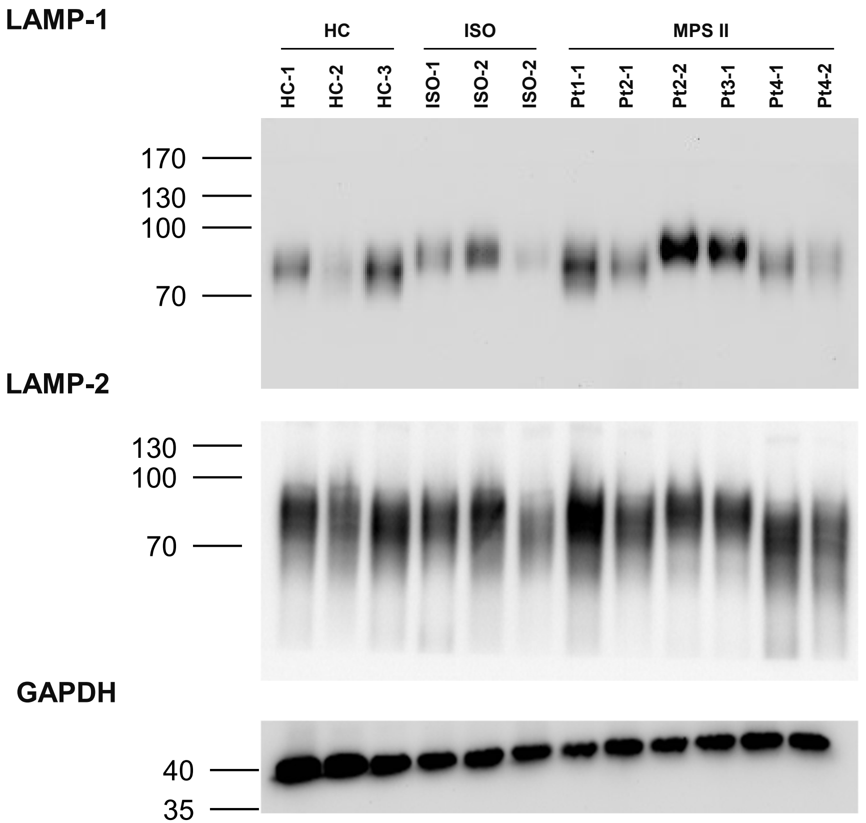

(Same membrane as p-GSK-3β)

original full-length Western blots presented in Supplementary Fig. 4B

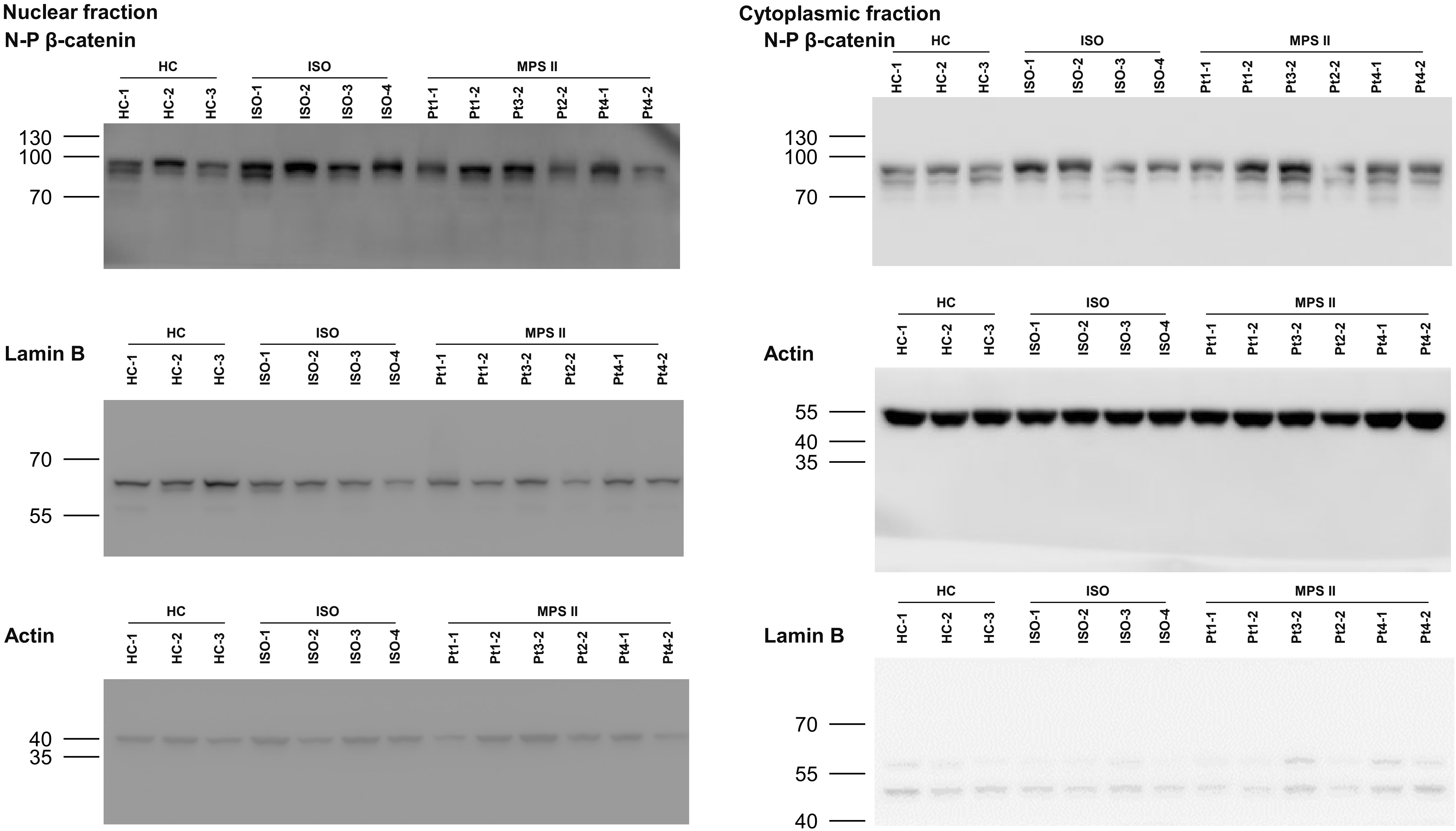

Additional blots for quantifications in Supplementary Fig. 4B: The green box indicates the protein of interest.

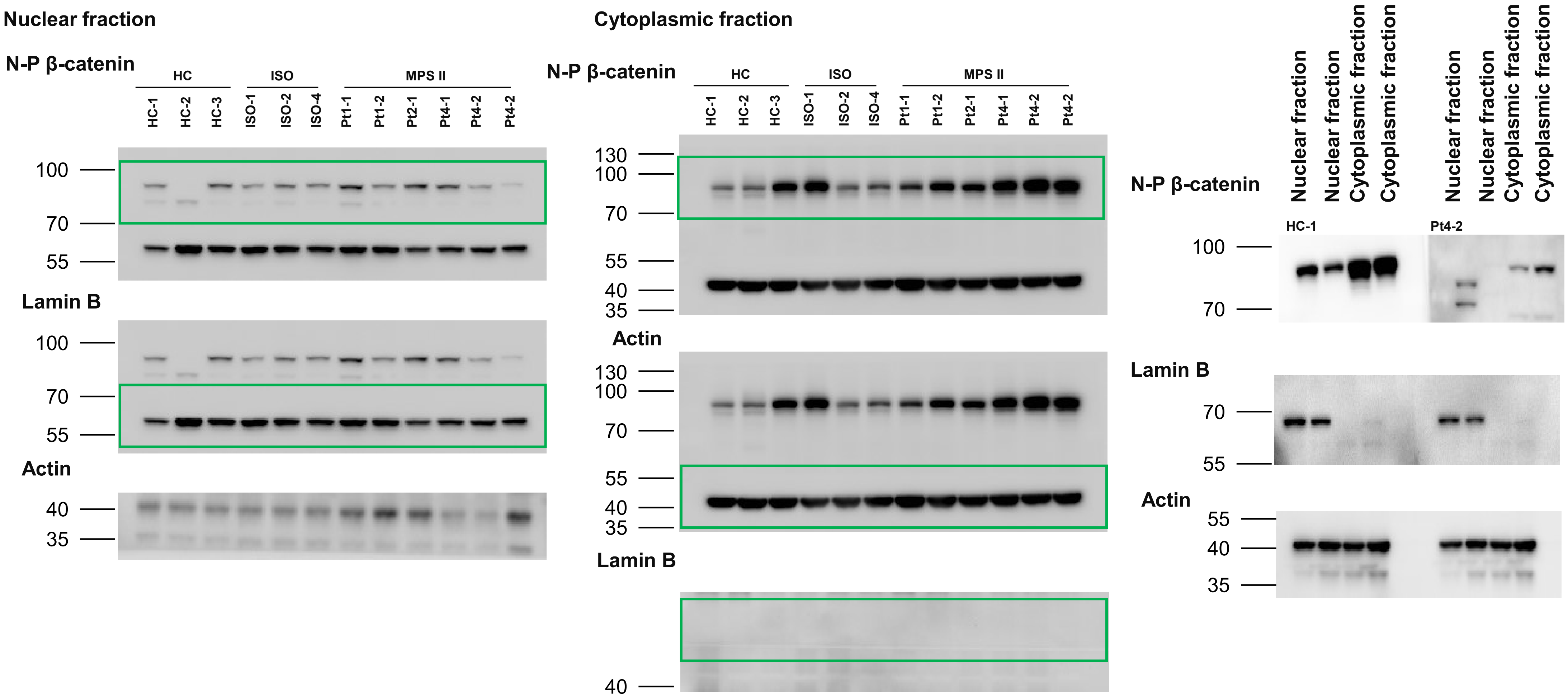

Supplement: Supplementary file 2 — original full-length Western blots [file 41419_2024_6692_MOESM2_ESM.pdf]
