# Supplementary material for: Mature neurons from iPSCs unveil neurodegeneration-related pathways in mucopolysaccharidosis type II: GSK-3β inhibition for therapeutic potential
Source: Cell Death Dis. 2024 Apr 29;15(4):302. doi: 10.1038/s41419-024-06692-9 (PMC11058230; doi:10.1038/s41419-024-06692-9)
Supplement: Supplementary file 3 — supplementary material [file 41419_2024_6692_MOESM3_ESM.pdf]

REAGENT or RESOURCE	SOURCE	IDENTIFIER
**Antibodies**		
Goat polyclonal NANOG	R&D	Cat# AF1997
Mouse monoclonal Tra-1-60	Chemicon	Cat# MAB4360
Mouse monoclonal Tuj1	BioLegend	Cat# 801201
Rabbit monoclonal Tuj1	Cell Signaling	Cat# 50-190-411
Rabbit polyclonal GFAP	Dako	Cat# Z0334
Rabbit polyclonal AFP	Dako	Cat# A0008
Mouse monoclonal LAMP-1	e-Bioscience	Cat# 14-1079-80
Rabbit polyclonal LAMP-2	e-Bioscience	Cat# 13555-T46
Rabbit polyclonal SOX2	Epitomics	Cat# s2683-S
Mouse monoclonal MAP2	Sigma-Aldrich	Cat# M4403
Mouse monoclonal Tau-1	Millipore	Cat# MAB3420
Mouse monoclonal Desmin	Dako	Cat# M0706
Rabbit polyclonal Cytokeratin 19	Abcam	Cat# ab15463
Mouse monoclonal GSK-3α/β	Santa Cruz	Cat# sc-7291
Mouse monoclonal phospho-GSK-3	Santa Cruz	Cat# sc-373800
Rabbit monoclonal Non-phospho β-catenin	Cell Signaling	Cat# 8814
Rabbit polyclonal Phospho-β-Catenin	Cell Signaling	Cat# 9561
Mouse monoclonal Lamin B	Santa Cruz	Cat# sc-365214
Rabbit polyclonal p38 MAPK (p38)	Cell Signaling	Cat# 9212
Rabbit polyclonal phospho-p38 MAPK (p-p38)	Cell Signaling	Cat# 9211
Rabbit monoclonal phospho-tau (Ser404)	Cell Signaling	Cat# 20194
Alexa Fluor® 594 Donkey anti-rabbit IgG	Biolegend	Cat# 406401
Alexa Fluor® 488 Donkey anti-rabbit IgG	Biolegend	Cat# 406416
Goat anti-Mouse IgG H+L Alexa Fluor 594	Invitrogen	Cat# A-11005
HRP Goat anti-mouse IgG	Biolegend	Cat# 405306
HRP Donkey anti-rabbit IgG	Biolegend	Cat# 406401
APC anti-GFP	Biolegend	Cat# 338009
LC3B (D11)	Cell Signaling	Cat# 3868
Nav1.7 Antibody (N68/6) (SCN9A)	Novus Biologicals	Cat#NBP2-12904
Kv4.2 Antibody (5B11B9) (KCND2)	Novus Biologicals	Cat# NBP2-52497
Anti-sloβ2 (KCNMB2)	Alomone	Cat#APC-034

NF-L Antibody (1H3) (NEFL)	Novus Biologicals	Cat# NBP2-37528
Ankyrin G	Santa Cruz	Cat#sc-12719
Spectrin	Santa Cruz	Cat#sc-53444
Recombinant PE Anti-Firefly Luciferase	Abcam	Cat# ab237253
**Chemicals and recombinant protein**		
A-443654	Cayman Chemical	Cat# 16499
Baicalein	Cayman Chemical	Cat#70610
Tideglusib	Cayman Chemical	Cat#16727
Odiparcil	MedChemExpress	Cat#HY-10277
Surfen	Tocris Bioscience	Cat# 6634
recombinant human HGF (NK1) protein	Qkine	Cat# Qk013
MSAB	Sigma-Aldrich	Cat# SML1726
Anisomycin	Cayman Chemical	Cat# 11308
SB203580	MedChemExpress	Cat# HY-10256
SB431542	Selleckchem	Cat# No.S1067
Heparan sulfate sodium salt from bovine kidney	Sigma-Aldrich	Cat#H7640
Dorsomorphin	Cayman Chemical	Cat# No. 11967
Basic Fibroblast Growth Factor (bFGF)	Thermo Fisher Scientific	Cat# No. PHG0264
**virus strains**		
AAV8-hSyn-EGFP	Addgene	#50465
pAAV-CaMKII α-hChR2(H134R)-EYFP	Addgene	#26969
pAAV-mDlx-GFP-Fishell-1	Addgene	#83900
**Critical commercial assays**		
CytoTune™-iPS 2.0 Sendai Reprogramming Kit	Thermo Fisher Scientific	Cat# A16517
Matrigel	Corning	Cat# 354234
Luciferase Assay System	PROMEGA	Cat# E1500

LEBT-M2	Moscerdam Substrate	N/A
GENEzol™ Reagent	Geneaid	Cat# GZR200
Iodoacetic acid	Sigma-Aldrich	Cat# 14386
SuperScript™ III Reverse Transcriptase	Thermo Fisher Scientific	Cat# 18080093
SYBR Green qPCR Master Mix	Thermo Fisher Scientific	Cat# 4309155
2X SuperRed MasterMix	BIOTOOLS Co., Ltd	Cat# TE-SR01
Vector® Blue Substrate Kit	Vector Laboratories	Cat# SK-5300
transIT-LT1 reagent	Mirus Bio	Cat# MIR 2300
Novagen® BCA Protein Assay Kit	Merck-Millipore	Cat# 71285-M
4-methylumbelliferyl-α-iduronate 2-sulfate	Moscerdam Substrates	
LEBT-M2	Moscerdam Substrates,	
XTT Cell Proliferation Assay Kit	Cayman Chemical	Cat# No. 10010200
1,9-dimethylmethylene blue	Sigma-Aldrich	Cat# 341088
Hoechst 33258	Sigma-Aldrich	94403
papain	Sigma-Aldrich	
Amersham™ ECL Select™ Western Blotting Detection Reagent	Cytiva	Cat# RPN2235
EthD-1	MyBioSource	Cat# mbs9718884
Calcein-AM	Biolegend	Cat# 425201
Protein G Sepharose 4 Fast Flow	Cytiva	Cat# 17061801
Fura-2, AM	Thermo Fisher Scientific	Cat# F1221
Thapsigargin	Thermo Fisher Scientific	Cat# T7459
Pluronic F127	Thermo Fisher Scientific	Cat# P3000MP
Ionomycin, Calcium Salt	Thermo Fisher Scientific	Cat# I24222
Probenecid	Thermo Fisher Scientific	Cat# P36400

Chloroquine (phosphate)	Cayman Chemical	Cat# No. 14194
(+/-)-Verapamil hydrochloride	Sigma-Aldrich	SI-V4629-1G
Proteinase K	Qiagen Inc.	Cat# 19131
**Experimental models: Organisms/strains**		
BALB/cAnN.Cg-Foxn1nu/CrlNarl	National Laboratory Animal Center	RMRC12005
**Oligonucleotides**		
**Endogenous**		
NANOG-F: AGTCCCAAAGGCAAACAACCCACTTC	this paper	N/A
NANOG-R: TGCTGGAGGCTGAGGTATTTCTGTCTC	this paper	N/A
OCT3/4-F: GACAGGGGGAGGGGAGGAGCTAGG	this paper	N/A
OCT3/4-R: CTTCCCTCCAACCAGTTGCCCCAAAC	this paper	N/A
SOX2-F: GGGAAATGGGAGGGGTGCAAAAGAGG	this paper	N/A
SOX2-R: TTGCGTGAGTGTGGATGGGATTGGTG	this paper	N/A
KLF4-F: ACGATCGTGGCCCCGGAAAAGGACC	this paper	N/A
KLF4-R: TGATTGTAGTGCTTTCTGGCTGGGCTCC	this paper	N/A
cMYC-F: GCGTCCTGGGAAGGGAGATCCGGAGC	this paper	N/A

cMYC-R: TTGAGGGGCATCGTCGCGGGAGGCTG	this paper	N/A
ACTB-F: GAGCACAGAGCCTCGCCTTT	this paper	N/A
ACTB-R: ACATGCCGGAGCCGTTGTC	this paper	N/A
GAPDH-F: CGGGAAACTGTGGCGTGATG	this paper	N/A
GAPDH-R: TGTGGAGGAGTGGGTGTCGCTGTT	this paper	N/A
**Virus titer**		
Lentiviral RRE-F: AGCTTTGTTCCTTGGGTTCTTGGGAG	this paper	N/A
Lentiviral RRE-R: AGGAGCTGTTGATCCTTTAGGTATCTTTCC	this paper	N/A
**Exogenous**		
SeV-F: GGATCACTAGGTGATATCGAGC	this paper	N/A
SeV-R: ACCAGACAAGAGTTTAAGAGATATGTATC	this paper	N/A
KOS-F: ATGCACCGCTACGACGTGAGCGC	this paper	N/A
KOS-R: ACCTTGACAATCCTGATGTGG	this paper	N/A
Klf4-F: TTCCTGCATGCCAGAGGAGCCC	this paper	N/A
Klf4-R: AATGTATCGAAGGTGCTCAA	this paper	N/A
c-Myc-F: GCGTCCTGGGAAGGGAGATCCGGAGC	this paper	N/A
c-Myc-R: TTGAGGGGCATCGTCGCGGGAGGCTG	this paper	N/A
**Patient mutation**		
Patient 1&2-IDS-F: ATATGGAGCCCAGACAGGTTC	this paper	N/A

Patient 1&2-IDS-R: CGACCAGCTCTAACTCCTCCT	this paper	N/A
Patient 3-IDS-F: CCGCCTCTTGAGTGCTTTGG	this paper	N/A
Patient 3-IDS-R: ATGCCCCAGGATCCCACTTT	this paper	N/A
Patient 4-IDS-F: GGTGGGCTCTAGGTGAACATGG	this paper	N/A
Patient 4-IDS-R: GGGGTCTGTGTAACCCCCAAA	this paper	N/A
**Neurons**		
NEFL-F: GGAAGAGGAGGCAGCTGAAGAG	this paper	N/A
NEFL-R: GTTCCTCCCCAGCACCTTCAA	this paper	N/A
GPC3 -F: GCACGATTGAACATGGAACAGC	this paper	N/A
GPC3 -R: GCATGGCGAACAACAATTTCAA	this paper	N/A
UPP-1-F: ACAAGCAGGCGTATCTGGAG	this paper	N/A
UPP-1-R: GTTCAGGAGGGTGACACACA	this paper	N/A
ABI-1-F: TGAGGAGGCTGCAGTAGTTC	this paper	N/A
ABI-1-R: TGCACCCTCCATAAATGACA	this paper	N/A
MMP7-F: GCAAAGAGATCCCCCTGCAT	this paper	N/A
MMP7-R: TGGCCCATCAAATGGGTAGG	this paper	N/A
FOSL1-F: AGTTACCCCAGGCCTCTGAC	this paper	N/A
FOSL1-R: CTGCAGCCCAGATTTCTCAT	this paper	N/A
AXIN2-F: AGGCCAGTGAGTTGGTTGTC	this paper	N/A
AXIN2-R: CATCCTCCCAGATCTCCTCA	this paper	N/A

cMYC-F: CTCTCCCGACGCGGGGAGGCTATTC	this paper	N/A
cMYC-R: TTGAGGGGCATCGTCGCGGGAGGCTG	this paper	N/A
OTX2-F: AGAGGAGGTGGCACTGAAAA	this paper	N/A
OTX2-R: ATTGGCCACTTGTTCCACTC	this paper	N/A
CCDC115-F: CCCTGAACTGGTTTGGAATC	this paper	N/A
CCDC115-R: TCTGGAGGCTGGCTATGTCT	this paper	N/A
SCN9A-F: TGTGTGCTAAAACTGATCTCCCTCA	this paper	N/A
SCN9A-R: GGGGACACAAAATACGTTTCAATCA	this paper	N/A
KCNMB2-F: TTCTTCCGGGGAAAAGCTCCT	this paper	N/A
KCNMB2-R: CAACATTCACCAGGGACATGGA	this paper	N/A
KCND2-F: CACGATTCAGATCAGATGTGTGGA	this paper	N/A
KCND2-R: TTACTGGAGGTGTTGGGATGCTT	this paper	N/A
NOTUM-F: AGTTCCAGGAGGGCGAGGAG	this paper	N/A
NOTUM-R: GCCTCGTCAAACAGCCACTG	this paper	N/A
WNT7B-F: GCGCCTCATGAACCTGCATA	this paper	N/A
WNT7B-R: GTTTGATGCGCAGGAAGGTG	this paper	N/A
RSPO3-F: CCTGTGTCCCCCAACAAATGA	this paper	N/A
RSPO3-R: CGTTGCTCTGGGATTTCTTTGC	this paper	N/A
FZD9-F: TCTTCCACATCCGCAAGATCA	this paper	N/A

FZD9-R: AGCAAACGATGACGCAGGTG	this paper	N/A
CANA1A-F: CGACGCCTGGACGATTACT	this paper	N/A
CANA1A-R: GGTCATGCTCAGGTCTGTCC	this paper	N/A
P2RX3-F: TCTCTGTGACATCATCCTGCTC	this paper	N/A
P2RX3-R: GAATCGGTGGACTGCTTCTC	this paper	N/A
ADRA1A-F: TCAGTGAGGCTCCTCAAGTTC	this paper	N/A
ADRA1A -R: AGGGCTTGAAATCAGGGAAG	this paper	N/A
ERBB4-F: CACGAGGAGAGCTGGATGA	this paper	N/A
ERBB4-R: GTTGGCAAAGGTGTTGAGGT	this paper	N/A
PLCD3-F: ACTCAGCCAACTACAGTCCC	this paper	N/A
PLCD3-R: CAAAGGTCGAGTCAGGTTGC	this paper	N/A
MYLK2-F: CTCAACAACCTGGCGGAGAA	this paper	N/A
MYLK2-R: CCCCGAGCTGCTGATCTT	this paper	N/A
PDE1B-F: TCAGCTTTCGTTCCACCTGG	this paper	N/A
PDE1B-R: CTGGTTGTGTTCATCTTCGGC	this paper	N/A
PLCB2-F: GTCGGAGAACGACACCAAAG	this paper	N/A
PLCB2-R: TGGTGCCTCTCCAAGTTCTC	this paper	N/A
RYR2-F: CATCTGTGGGATAGGCAATG	this paper	N/A
RYR2-R: GGGAAAAATTCCCAACACCT	this paper	N/A
**Chip assay**		

SCN9A-1-F: AGTGGTCAGTTTCTGTTGGC	this paper	N/A
SCN9A-1-R: TCAATGAAAAATTTGAAAAAGTACAGA	this paper	N/A
SCN9A-2-F: CAGGATGAAAAGATGGCAATG	this paper	N/A
SCN9A-2-R: AAGTCACTGCTTGGCTTTGG	this paper	N/A
SCN9A-3-F: AAGATAAGTCCCGCCCATTG	this paper	N/A
SCN9A-3-R: TATTTGCCTGCCAAGAAAGG	this paper	N/A
SCN9A-4-F: TGACTGAATTGCTGCCATCT	this paper	N/A
SCN9A-4-R: CCTCTGCTGTCACTTCAACAA	this paper	N/A
SCN9A-5-F: GAACTGCAGATGTGGTGGAA	this paper	N/A
SCN9A-5-R: TCCATCTCACGAAACCACTTT	this paper	N/A
**Software and algorithms**		
FlowJo	BD Biosciences	X10 https://www.flowjo.com/
FACSCalibur™ Flow Cytometer	BD Biosciences	E3017
GraphPad Prism 8	Dotmatics	https://www.graphpad.com/features
EditR	Kluesner, M. G et.al, 2018	EditR 1.0.10 https://moriaritylab.shinyapps.io/editr_v10/
ZEISS Axio Observer 7	Carl ZEISS	Axio Observer 7
ABI QuantStudio 5	Thermo Fisher Scientific	A34322
ImageJ	National Institutes of Health	https://imagej.nih.gov/ij/download.html
Fiji	National Institutes of Health	https://fiji.sc/

Clampfit	Molecular Devices	Clampfit 10.6
Bioruptor Plus sonication device	Diagenode	Cat# B01020001
SpectraMax i3x Multi-Mode Microplate reader	Molecular Devices	https://www.moleculardevices.com/products/microplate-readers/multi-mode-readers/spectramax-i3x-readers
Axio Examiner D1	Carl ZEISS	
Multiclamp 700 B	Molecular Devices	
NEUROBIOTIN Tracer	Vector Laboratories, Inc.	SP-1120
Neurolucida software	MBF Bioscience	
FUSION Solo S	Vilber	
CellQuest software	BD Biosciences	
BD FACSAria™ III | High Sensitivity Flow Cytometer	BD Biosciences	
NIS-Elements Viewer	Nikon	5.21
